# Giant viruses of the *Megavirinae* subfamily possess biosynthetic pathways to produce rare bacterial-like sugars in a clade-specific manner

**DOI:** 10.1093/femsml/uqac002

**Published:** 2022-04-06

**Authors:** Anna Notaro, Olivier Poirot, Elsa D Garcin, Sebastien Nin, Antonio Molinaro, Michela Tonetti, Cristina De Castro, Chantal Abergel

**Affiliations:** University of Naples Federico II, Department of Agricultural Sciences, Via Università100, 80055, Portici, Naples, Italy; Aix-Marseille University and Centre National de la Recherche Scientifique and Institut de Microbiology de la Méditerranée; IGS Unité Mixte de Recherche 7256, FR3479, IM2B, 13288 Marseille Cedex 9, France; Aix-Marseille University and Centre National de la Recherche Scientifique and Institut de Microbiology de la Méditerranée; IGS Unité Mixte de Recherche 7256, FR3479, IM2B, 13288 Marseille Cedex 9, France; Aix-Marseille University and Centre National de la Recherche Scientifique and Institut de Microbiology de la Méditerranée; IGS Unité Mixte de Recherche 7256, FR3479, IM2B, 13288 Marseille Cedex 9, France; Aix-Marseille University and Centre National de la Recherche Scientifique and Institut de Microbiology de la Méditerranée; IGS Unité Mixte de Recherche 7256, FR3479, IM2B, 13288 Marseille Cedex 9, France; Department of Chemical Sciences, University of Naples Federico II, Via Cinthia 26, 80126, Naples, Italy; Department of Experimental Medicine and Center of Excellence for Biomedical Research, University of Genova, Viale Benedetto XV, 1, 16132, Genova, Italy; University of Naples Federico II, Department of Agricultural Sciences, Via Università100, 80055, Portici, Naples, Italy; Aix-Marseille University and Centre National de la Recherche Scientifique and Institut de Microbiology de la Méditerranée; IGS Unité Mixte de Recherche 7256, FR3479, IM2B, 13288 Marseille Cedex 9, France

**Keywords:** giant virus, proposed *Megavirinae* subfamily, viral glycosylation, chemical analysis, bioinformatics

## Abstract

The recent discovery that giant viruses encode proteins related to sugar synthesis and processing paved the way for the study of their glycosylation machinery. We focused on the proposed *Megavirinae* subfamily, for which glycan-related genes were proposed to code for proteins involved in glycosylation of the layer of fibrils surrounding their icosahedral capsids. We compared sugar compositions and corresponding biosynthetic pathways among clade members using a combination of chemical and bioinformatics approaches. We first demonstrated that *Megavirinae* glycosylation differs in many aspects from what was previously reported for viruses, as they have complex glycosylation gene clusters made of six and up to 33 genes to synthetize their fibril glycans (biosynthetic pathways for nucleotide-sugars and glycosyltransferases). Second, they synthesize rare amino-sugars, usually restricted to bacteria and absent from their eukaryotic host. Finally, we showed that *Megavirinae* glycosylation is clade-specific and that *Moumouvirus australiensis*, a B-clade outsider, shares key features with *Cotonvirus japonicus* (clade E) and *Tupanviruses* (clade D). The existence of a glycosylation toolbox in this family could represent an advantageous strategy to survive in an environment where members of the same family are competing for the same amoeba host. This study expands the field of viral glycobiology and raises questions on how *Megavirinae* evolved such versatile glycosylation machinery.

## Teaser

The fibrils decorating the viral capsids of members of the *Megavirinae* are glycosylated by clade-specific machineries.

## Introduction

**Figure 1.**
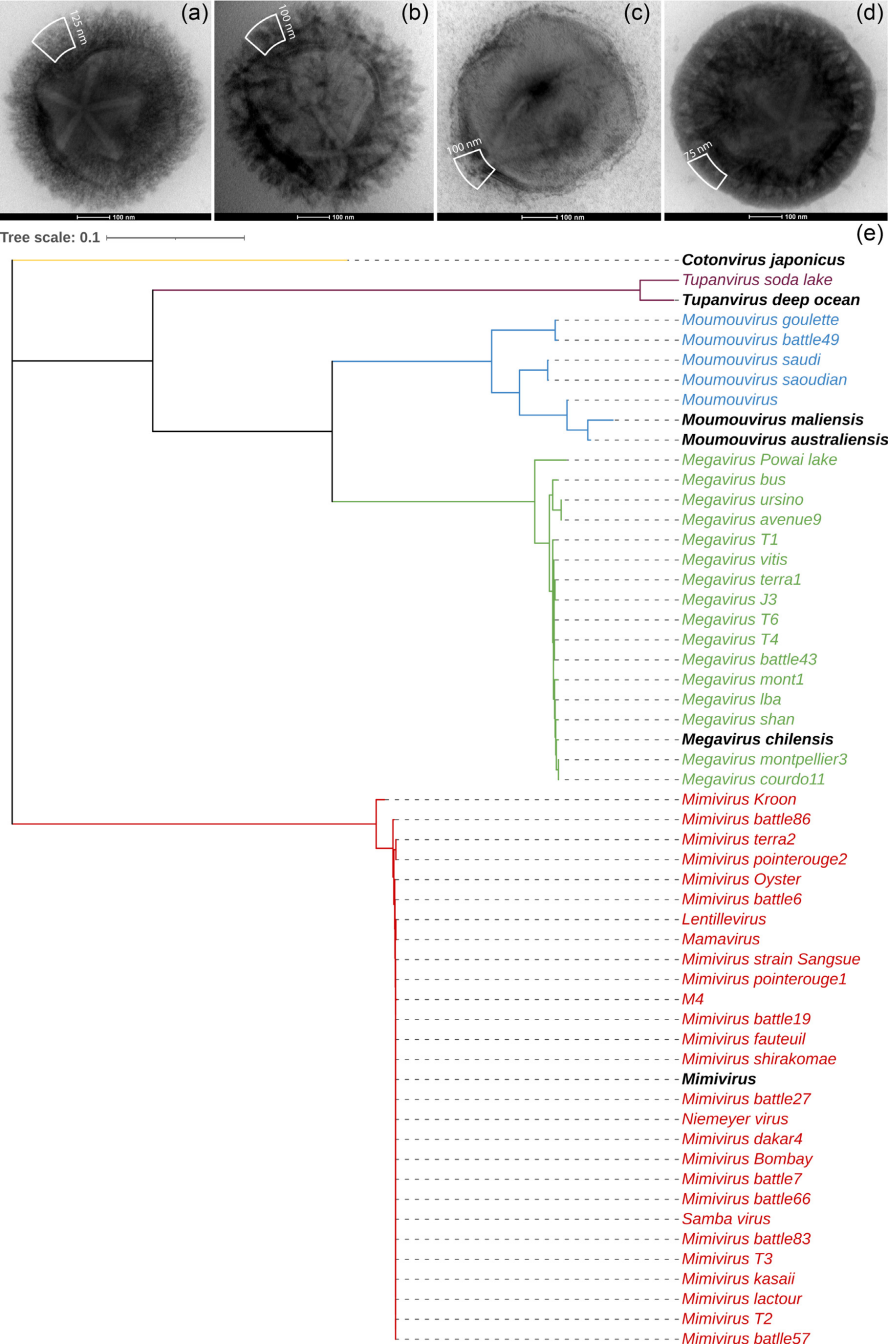
Top: Transmission electron microscopy (TEM) images of negative-stained (NS) virions of the proposed *Megavirinae*subfamily. **(a)***Mimivirus* (A-clade); **(b)***Moumouvirus maliensis* (B-clade); **(c)***Moumouvirus australiensis* (B-clade); (**d)***Megavirus chilensis* (C-clade). White boxes delineate the fibril layers. Thickness of the layer was measured on TEM images of resin-embedded infected cells when mature particles were visible, as NS-TEM on dehydrated samples can induce fibril collapse or shrinking; **(e)**phylogenetic tree of the proposed *Megavirinae* subfamily. This family encompasses five clades: *Mimiviruses* (A-clade, in red), *Moumouviruses* (B-clade, in blue), *Megaviruses* (C-clade, in green), *Tupanviruses* (D-clade, in violet) and *Cotonvirus* (E-clade, in yellow). For each clade, the prototypes are in black and in bold. The tree is based on the concatenation and alignment of seven protein markers (see Methods).

The general perception of viruses as small and simple entities has been challenged with the discovery of giant viruses (B. La Scola *et al*. 2003, Abergel *et al*. 2015). Giant viruses are endowed with dsDNA genomes up to 2.5 Mb that can encode up to 1500 proteins while more conventional viruses have much smaller genomes and sometimes just a handful of genes (Lu *et al*. 2020). Giant virus capsids are so large (up to 2 µm) that they can easily be seen by light microscopy. These viruses are larger than the smallest bacteria (*Mycoplasma genitalium* <0.3 μm) and archaea (*Nanoarchaeum equitans*, 0.4 μm) and contain more genes than the smallest parasitic eukaryote encephalitozoon (Philippe *et al*. 2013). They all infect unicellular eukaryotes and are major players in the environment (Suttle 2005, Brussaard *et al*. 2008) where they regulate protist populations. Given their genomic complexity, they contain genes never encountered before in viruses (B. La Scola *et al*. 2003, Renesto *et al*. 2006), such as those related to protein translation (Abergel *et al*. 2007, Raoult 2004, Jeudy *et al*. 2012) and glycan synthesis (Parakkottil Chothi *et al*. 2010, Piacente *et al*. 2012, 2014a,b,2017a). Currently, several families of giant viruses have been discovered, such as the *Mimiviridae* and the proposed *Pandoraviridae*, *Molliviridae* and *Pithoviridae* (Abergel *et al*. 2015). The present study is focused on the *Mimiviridae* family and in particular on the proposed *Megavirinae* subfamily (Gallot-Lavallée *et al*. 2017), which encompasses five clades, all infecting *Acanthamoeba*sp. (Fig. 1): Mimiviruses (A-clade), Moumouviruses (B-clade), Megaviruses (C-clade), Tupanviruses (D-clade) (Abrahão *et al*. 2018) and the recently isolated *Cotonvirus japonicus* (E-clade) (Takahashi *et al*. 2021). As of today, all members of the proposed *Megavirinae* are characterized by a fibril layer that differs in thickness and length among the clades, as shown by negative staining transmission electron microscopy (NS-TEM) (Fig. 1). Interestingly, NS-TEM images of *Moumouvirus australiensis* (Fig. 1c) and *Moumouvirus maliensis* (Fig. 1b) also presented marked differences in their fibril layer thickness while supposedly belonging to the same clade.

**Figure 2.**
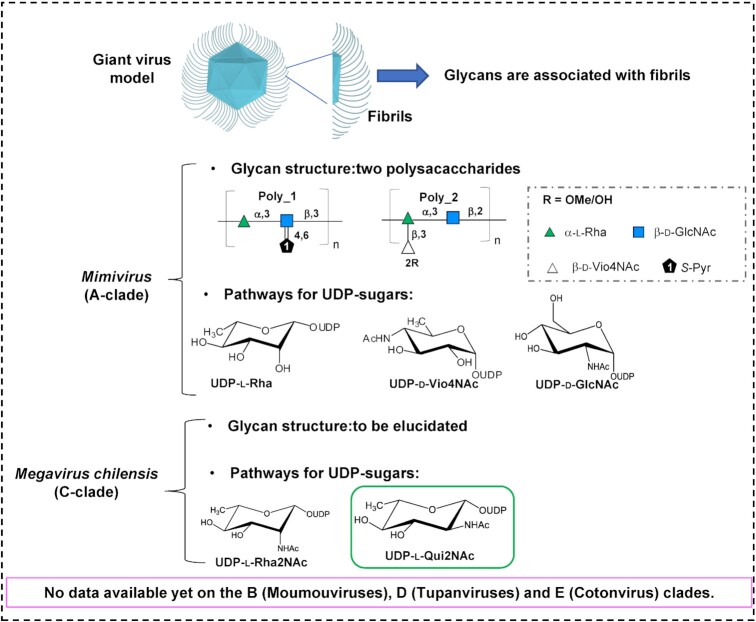
Glycosylation in the proposed *Megavirinae* subfamily. A hallmark of the members of the family is the presence of a fibril layer surrounding the icosahedral capsids with evidence for some of the associated sugars. The glycan structure of *Mimivirus* (A-clade) has been elucidated: poly_1 has a linear repeating unit made of 3)-α-l-Rha-(1→3)-β-d-GlcNAc-(1→, with GlcNAc modified by pyruvic acid at position 4,6; poly_2 has a branched repeating unit made of 2)-α-l-Rha-(1→3)-β-d-GlcNAc-(1→ in the linear backbone; Rha is further branched with a terminal β-d-2OMeVio4NAc, which is 75% methylated. The biosynthetic pathways for UDP-l-rhamnose (UDP-l-Rha), UDP-d-N-acetyl-glucosamine (UDP-d-GlcNAc) and UDP-d-4N-acetyl-viosamine (UDP-d-Vio4NAc) have been predicted and experimentally validated. For *Megavirus chilensis* (C-clade) amino-sugars are found on the fibrils, but the glycan structure is still unknown. The biosynthetic pathways for UDP-l-rhamnosamine (UDP-l-Rha2NAc) and UDP-l-quinovosamine were predicted (UDP-l-Qui2NAc, green box), but experimental validation was only performed for the UDP-l-Rha2NAc pathway. No data are available for the other clades.

Recent studies on *Mimivirus* (A-clade) revealed that complex glycans with unique structures made of two large polysaccharides were branched to the fibrils (Fig. 2). These carbohydrate polymers are made of up to 20 units of sugars that are not synthetized by the amoeba host (Notaro *et al*. 2021). This result challenged the common belief that eukaryotic viruses divert the host machinery to decorate their envelope proteins with small oligosaccharides containing two to 10 sugar units (Bagdonaite and Wandall 2018). For *Megavirus chilensis* (C-clade) virions, preliminary analysis revealed that its fibrils were also composed of rare amino-sugars not synthesized by the amoeba host (Piacente *et al*. 2014b).

Similarly, in PBCV-1, a large dsDNA virus of the *Phycodnaviridae* family, the Major Capsid Protein was shown to be decorated with an oligosaccharide that was different from those found in the three domains of life (De Castro *et al*. 2013). The unique nature of giant viruses glycans compared to their hosts is made possible by the presence of genes involved in glycosylation in their complex genomes. In PBCV-1, two biosynthetic pathways for sugars activated as nucleotides (donor-sugars) have been found (Piacente *et al*. 2015), along with a great number of predicted and experimentally validated glycosyltransferases (Noel *et al*. 2021, Speciale *et al*. 2020). Similarly, for the proposed *Megavirinae* subfamily, biosynthetic pathways for nucleotide-sugars along with glycosyltransferases (Fig. 2) have been discovered for *Mimivirus* and *Megavirus chilensis* (Parakkottil Chothi *et al*. 2010, Piacente *et al*. 2012, 2014a,b, 2017a). Functional pathways for UDP-l-rhamnose (UDP-L-Rha), UDP-d-glucosamine (UDP-d-GlcNAc) and UDP-d-4N-acetyl-viosamine (UDP-d-Vio4NAc) in *Mimivirus* (Parakkottil Chothi *et al*. 2010; Piacente *et al*. 2012, 2014a,b,2017a) and a synthesis pathway for UDP-l-rhamnosamine (UDP-l-Rha2NAc) in *Megavirus chilensis* (Piacente *et al*. 2014b) have been experimentally validated. However, none of the predicted glycosyltransferases (GT) have been experimentally characterized, and there are no data available on glycans and glycogenes for the other clades of the family (Fig. 2).

A global view of glycosylation in the proposed *Megavirinae* subfamily is a prerequisite to make progress in the nascent field of viral glycosylation and increase our knowledge on carbohydrates and their biosynthesis. Key questions include (i) What is the viral machinery (glycogenes) used to synthesize these complex sugars in the different clades of the proposed *Megavirinae* subfamily? (ii) What is the nature of the glycans synthesized by other members of this subfamily? (iii) Do these glycans share a common architecture, as seen in *Chloroviruses* (De Castro *et al*. 2016), or is their nature and architecture clade specific? To answer these questions, the present study aimed at comparing glycosylation of the giant viruses of the proposed *Megavirinae* subfamily by exploring the composition of their glycans and searching their genomes to identify possible genes involved in their biosynthesis.

To do this, we combined carbohydrate chemistry with bioinformatic analyses for members of each clade. *Mimivirus* (Scola 2003) and *Megavirus chilensis* (Arslan *et al*. 2011) were used as prototypes for the A- and C-clades, respectively, while we used both *Moumouvirus australiensis* and *maliensis* for the B-clade. For each, we chemically characterized their glycan composition, and performed an *in silico* comparative analysis to evaluate similarities/differences within each clade and within the entire family. As dense fibrils also decorate *Tupanviruses* (D-clade) and *Cotonvirus japonicus* (E-clade), we searched their genomes for putative glycogenes and included these data to provide a comprehensive comparative analysis covering all members of the family.

## Methods

### Phylogeny of the proposed *Megavirinae* subfamily

The phylogenetic tree of the proposed *Megavirinae* subfamily is based on concatenation and alignment of the following seven protein markers: Asp-Synthase, Helicase, mRNA Capping Enzyme, MutS, Packaging ATPase, PolyA polymerase and VLTF3. Fifty-five genomes of members of the different clades were used to generate the phylogenetic tree using the command line: mafft (v7.307) for the alignment, trimal (v1.4. rev22) for the gap filtering and iqtree (v1.6.9), with default settings at each step.

### Production and purification of the virions

All viruses described here as prototypes of the different clades have been isolated by our laboratory. The protocol adopted to propagate and purify the viral particles is the same for all members of the different clades. Briefly, viral particles were propagated using *A. castellanii* (Douglas) Neff (American Type Culture Collection 30010TM) cells, which were infected with viruses at a multiplicity of infection of 0.25. After 2 days of incubation at 32°C, the infection was complete and led to cell lysis and release of viral particles into the culture medium. Viral particles were then purified by removing cell debris by centrifugation at 500 g for 10 min at 20°C. Subsequently, the supernatant containing viral particles was spun at 6,800 g, 45 min at 20°C. The pellet containing the virions was washed with water, resuspended in CsCl 1.2 density, deposited on a discontinuous CsCl gradient made of successive layers of 1.3/1.4/1.5 densities (g/ml) and finally spun for 20 h at 100 000 g. The white band corresponding to viral particles was recovered with a syringe and washed three times with water. Purified virions were imaged by light microscopy (ZEISS) and the concentration of the virion was estimated on a spectrophotometer (Eppendorf) at OD 600 nm.

### NS-TEM of the virions

Viral particles were visualized by NS-TEM, as reported previously (Notaro *et al*. 2021). Briefly, viral particles were fixed in glutaraldehyde (2.5% v/v in water) for 1 h at room temperature. Samples were centrifuged at 5000 g for 10 min and pellets were washed twice with water. The structure of the fibrils was visualized by NS-TEM using methyl cellulose (M6385 Sigma) and uranyl acetate (2% v/v in water) as reported (Notaro *et al*. 2021). Viral particles were observed by TEM on a TECNAI G2°200 KV.

### Sugar composition

Monosaccharide composition analysis (as acetylated methyl glycoside) and determination of their absolute configuration (as octyl-glycosides derivatives) was performed on 1×10^10^ viral particles, as previously reported (De Castro *et al*. 2010). Gas chromatography-mass spectrometry (GC-MS) analyses were performed on an Agilent instrument (GC instrument Agilent 6850 coupled to MS Agilent 5973) equipped with a SPB-5 capillary column (Supelco, 30 m × 0.25 i.d., flow rate, 0.8 mL min^–1^) and He as the carrier gas. Electron impact mass spectra were recorded with an ionization energy of 70 eV and an ionizing current of 0.2 mA. The temperature program used for analyses was as follows: 150°C for 5 min, 150 to 280°C at 3°C/min, 300°C for 5 min. Interpretation of these data is based on the following concept: each sugar that is derivatized as acetylated methyl-glycoside or octyl-glycoside is eluted in a specific range of the chromatogram and each peak corresponds to a specific fragmentation pattern, which enables identification of the monosaccharide (Lönngren and Svensson 1974). Elution time is compared with those of standard monosaccharides and allows discrimination between sugars of the same class (i.e. glucose and mannose) and between sugars with different absolute configuration (D or L).

### Identification of new genes encoding proteins involved in fibril glycosylation for each clade

We used an *in silico* approach to search for genes coding for potential proteins involved in glycosylation, hereafter referred to as glycogenes. Because giant virus genetics was not yet available, we could not validate these predictions by mutagenesis. Instead, we considered that identification of predicted sugars by chemical analysis of the viral particles validated the presence of a functional pathway encoded by the virus, because the amoeba host does not synthesize these rare bacteria-like sugars. The general approach used to attribute a specific function to a gene consisted in the comparison of the translated sequences with reference annotated protein sequences by a multiple alignment based on structural information, using the Expresso Server (Armougom *et al*. 2006) (http://tcoffee.crg.cat/apps/tcoffee/do:expresso). Multiple alignments were submitted to the ESPript server (http://espript.ibcp.fr/ESPript/ESPript/) to illustrate sequence similarities and display secondary structure elements (Gouet *et al*. 2003).

Multiple alignments allowed to verify if catalytic residues were conserved in candidate proteins compared with the reference protein for which the function and structure were known. Conservation of the catalytic site was a prerequisite to attribute a specific function. In addition, we used the HHpred server (Hildebrand *et al*. 2009) for remote homology detection to annotate hypothetical proteins. We only kept those for which the confidence level was above 99%.

To understand if identified glycogenes were recently acquired from other microorganisms, such as bacteria on which the amoeba feeds, we compared the GC content of these genes with those of the seven marker genes (Asp Synthase, Helicase, mRNA Capping Enzyme, MutS, Packaging ATPase, PolyA polymerase and VLTF3) as representatives of whole genome GC content. GC content was computed using geecee (Emboss package v6.6.0, https://www.bioinformatics.nl/cgi-bin/emboss/geecee).

### Conservation of the proteins involved in fibril glycosylation in the proposed *Megavirinae*

Tblastn was used to assess the presence and conservation level of all proteins possibly involved in fibril glycosylation in all members of the three clades. We restricted the study to those for which complete genome sequences were available in the NCBI database (Table S1). Results are presented as conservation heatmaps, which were built using an in-house developed software. Construction of the heatmap was based on the following steps:

1. tblastn of N proteins (protein file) against M genomes (genome file), so that each protein sequence (query) is compared with the six-frame translations of nucleotide genome sequences;
2. for each protein, recovery of the best scoring ORF in each genome, with normalization by the size of the protein (score/autoscore), which defines the ‘conservation score’ in heatmaps;
3. generation of the matrix (N best scores) x (M genomes) and plot of the heatmap (library pheatmap in R, with default parameters).

NCBI accession numbers of complete genome sequences and of protein sequences (query) are reported in Tables S1 and S2, respectively.

## Results

### Sugar composition varies in fibrils from viruses in different clades

**Figure 3.**
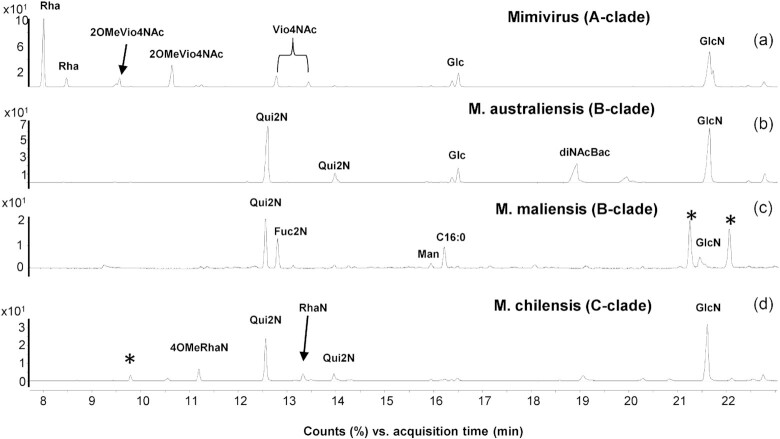
Sugar composition of the fibrils for members of the proposed *Megavirinae* subfamily by GC-MS. Chromatogram profiles of the sugars composing the fibrils of (**a**) *Mimivirus* (Notaro *et al*. 2021), (**b**) *Moumouvirus australiensis*, (**c**) *Moumouvirus maliensis* and **(d**) *Megavirus chilensis*. Known standards have been used to assign peaks to the corresponding monosaccharides. The * indicates impurities.

*Mimivirus* (A-clade), *Moumouvirus australiensis* and *maliensis* (B-clade) and *Megavirus chilensis* (C-clade) were used as prototypes to investigate the glycan composition of their fibrils and identify conserved and clade-specific features. The nature of two complex polysaccharides (Fig. 2) composing *Mimivirus* fibrils has recently been characterized (Notaro *et al*. 2021), and they involve three different sugar moieties: rhamnose (Rha), N-acetyl-glucosamine (GlcNAc) and N-acetyl-viosamine (Vio4NAc) (Fig. 3a). Here, we characterized all sugar moieties constituting the fibrils of representative members of B- and C-clades.

First, chemical characterization of B-clade *Moumouvirus australiensis* and *maliensis* fibrils revealed different sugar compositions. Fibrils from *Moumouvirus australiensis* (Jeudy *et al*. 2020) contained glucosamine (GlcN), quinovosamine (Qui2N) and bacillosamine (diNAcBac) as major components and glucose (Glc) as a minor component (Fig. 3b). The peak at 19 min was identified as diNAcBac by applying the fragmentation rules of the acetylated methylglycosides derivatives (Lönngren and Svensson 1974); indeed, the EI-MS spectrum contained a fragment at m/z 271 consistent with the oxonium ion of a six-deoxy-sugar with two amino functions (Fig. S1).

By contrast, fibrils from *Moumouvirus maliensis* contained fucosamine (Fuc2N) and Qui2N as major components, while mannose (Man) and GlcN were present in small amounts (Fig. 3c).

Previous analysis performed on intact virions from *Megavirus chilensis*, the prototype of C-clade, had revealed the presence of GlcN, rhamnosamine (RhaN) and RhaN methylated at position 4 (4-OMe-RhaN) as the main components of the fibrils together with an unidentified rhamnosamine epimer (Piacente *et al*. 2014b). Here, we completed this analysis and identified the nature of this component as Qui2N, in agreement with previous results revealing the biosynthetic pathways encoded by the virus (Piacente *et al*. 2014b). In addition, our current analysis confirmed the presence of GlcN, RhaN and 4-OMe-RhaN (Fig. 3d).

We determined absolute configurations for most sugars for members of all clades. The absolute configuration of GlcN was experimentally confirmed to be d for *Mimivirus* (Fig. S2), in agreement with the presence of a biosynthetic pathway for UDP-d-GlcNAc (Piacente *et al*. 2014a). Similarly, we showed that glycans in *Moumouvirus australiensis* and *Megavirus chilensis* contained d-GlcNAc (Fig. S2), and that Qui2N was in the l configuration. By contrast, we determined that Qui2N and Fuc2N were both in the d configuration for *Moumouvirus maliensis* (Fig. S2). We could not determine the absolute configuration of Rha2N and diNAcBac due to the lack of appropriate standards. However, it is likely that the absolute configuration of Rha2N is l, because the UDP-l-Rha2NAc biosynthetic pathway was validated *in vitro* for *Megavirus chilensis* (Piacente *et al*. 2014b). In bacteria, diNAcBac is in the d configuration (Morrison and Imperiali 2014), suggesting that this sugar could adopt the same configuration in *Moumouvirus australiensis* glycans.

### Filling the gap between experimental and genomic data for *Mimivirus* (A-clade)

**Figure 4.**
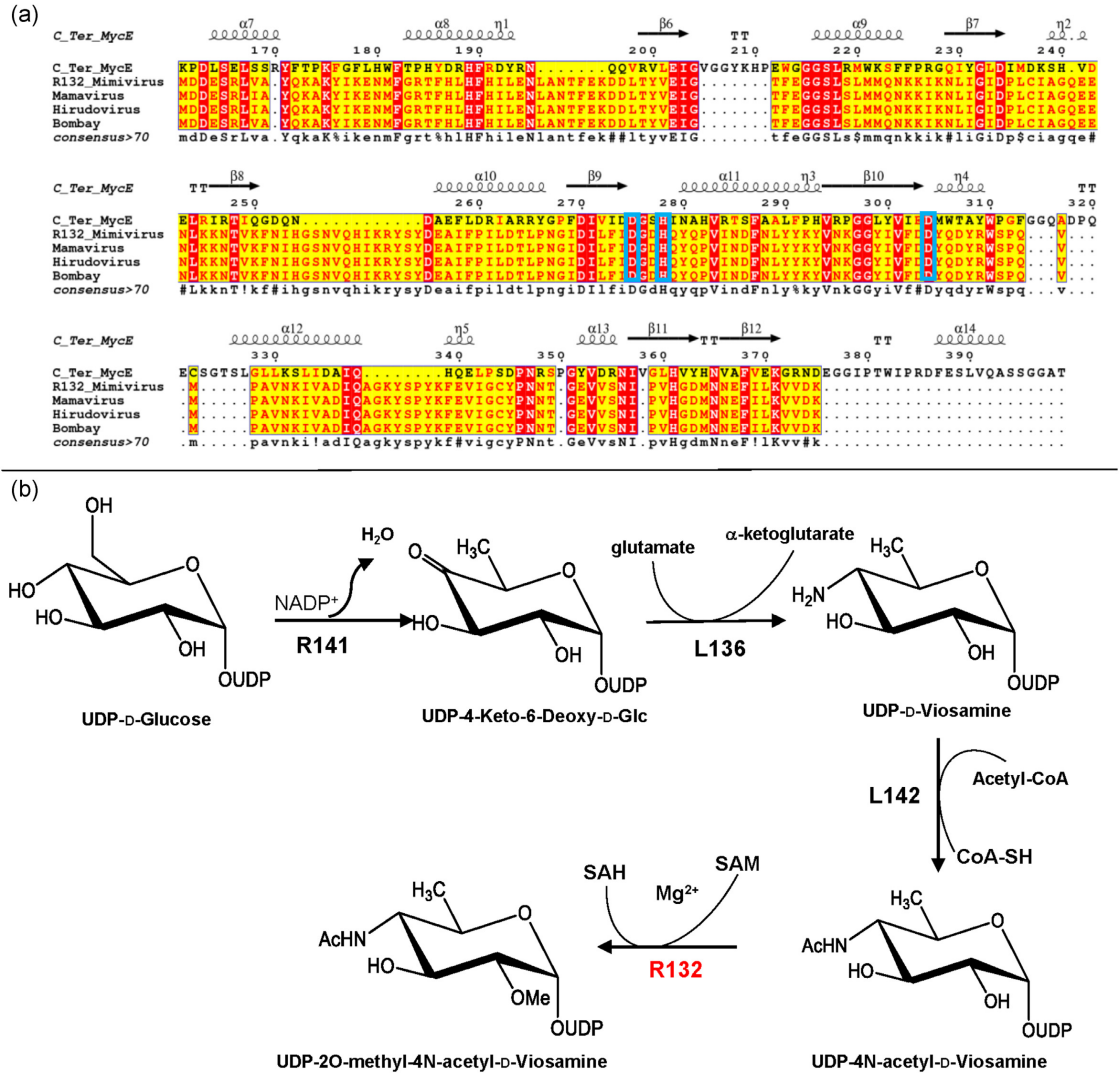
R132 methyltransferase, a candidate for viosamine methylation. **(a)** Multiple alignment of the C-Ter of MycE (PDB 3SSN), R132 of *Mimivirus* (YP_003 986 624), its homolog in *Mamavirus* (AEQ60310.1)*, Hirudovirus* strain Sangsue (AHA45742.1) and *Mimivirus* Bombay (AMZ02580.1). The blue rectangles indicate the residues involved in catalytic activity: the two aspartates coordinating the magnesium D275 and D304 (Akey *et al*. 2011) are conserved in R132 and A-clade (D127 and D156); the histidine residue (H278) involved in deprotonation of the 2-OH is also conserved (H138). **(b)** Proposed biosynthetic pathway for the UDP-d-2O-methyl-4N-acetyl-viosamine. The functions of the first three enzymes have been experimentally validated and R132, based on *in silico* analysis, would be the missing methyltransferase.

The elucidation of the glycan structures of *Mimivirus* fibrils (Fig. 2) (Notaro *et al*. 2021) prompted the search for genes coding for proteins that could be involved in the biosynthesis of these polysaccharides. A previous study had proposed a nine-gene cluster (Piacente *et al*. 2012) that includes genes encoding proteins necessary for the biosynthesis of Vio4NAc (*R141*, *L136*, *L142*), a gene annotated as pyruvyltransferase (*L143*), several genes encoding glycosyltransferases (*L137*, *L138*, *R139*, *L140* and the C-ter of L142) and a gene encoding a GMC-type oxidoreductase (*R135*). However, a gene coding for a Vio4NAc methyltransferase was missing, raising the question on the origin of this sugar modification. Viosamine is not produced by the amoeba host and is only encountered in bacteria. For example, *Pseudomonas syringe* possesses a Vio-island including all the genes involved in viosamine production, methylation and transfer (Yamamoto *et al*. 2011). Thus, we hypothesized that a similar organization could exist in the *Mimivirus* genome. We narrowed the search within 2 kbp of the nine-gene cluster (Piacente *et al*. 2012, 2017b) and identified *R132* as a promising candidate. The *R132* gene is predicted to encode a 221-amino-acid protein belonging to class I SAM-dependent methyltransferases. Its closest homologs are bacterial methyltransferases from *Rizhobiales* (WP_112 557 168.1), *Lelliottia* (WP_107 702 876.1) and *Pantoea dispersa* (WP_021 509 887.1), which share 35 to 39% sequence identity with R132 on the entire length of the protein sequence. The R132 protein was modeled using Phyre2 with 100% confidence based on several sugar-methyltransferases structures and confirmed by AlphaFold (Jumper *et al*. 2021). The best ranked model was obtained using the C-terminal catalytic domain of MycE as template (residues 161–399, 20% sequence identity). This SAM and metal-dependent methyltransferase domain is responsible for methylation of the 6-deoxyallose sugar moiety of mycinamicins (Akey *et al*. 2011). A multiple-alignment of R132 and its orthologs in A-clade members with the C-terminal domain of MycE (Fig. 4a) revealed that all catalytic residues are conserved, suggesting that *R132* could encode a functional methyltransferase (Fig. 4a). Further support of this prediction comes from two additional observations. First, previous studies showed that timing and expression level of *R132* were comparable with those of the nine genes belonging to the glycan formation cluster (Legendre *et al*. 2010). Second, the *R132* gene is absent from the genome of *Mimivirus* M4. This virus is the result of a 150-times subculture of *Mimivirus* in a germ-free amoeba host that led to the dramatic reduction of its genome from 1.20 to 0.993 Mbp due to two large deletions, mainly at the two extremities of the genome. One of these deletions includes genes encoding for proteins known to be involved in sugar biosynthesis (Boyer *et al*. 2011a, Piacente *et al*. 2012) as well as structural proteins composing the fibrils such as GMC-oxidoreductase R135 (Klose *et al*. 2015). Taken together, our results suggest a possible role for R132 in O-2 methylation of Vio4NAc. We thus propose that *R132* is part of the gene cluster encoding a biosynthetic pathway for the sugars of structural proteins making *Mimivirus* fibrils.

As proposed in a previous study, the methylation reaction should occur after the acetylation of the amino function at position 4 (Fig. 3b), due to the steric hindrance caused by the 2OMe on the viosamine moiety that would impair acetylation by the L142 enzyme (Piacente *et al*. 2017b). We still do not know if methylation occurs on the UDP-sugar, as reported in Fig. 4 or on viosamine inside the polysaccharide chain, this point being only addressable experimentally.

### Biosynthetic pathways for nucleotide-sugars in *Moumouvirus australiensis*(B-clade)

Here, we identified the monosaccharides composing *Moumouvirus australiensis* fibrils (Fig. 3b) as GlcN, Qui2N and diNAcBac. To determine if its genome encodes proteins necessary for biosynthesis of these sugars as activated nucleotides, we searched for genes and proteins similar to those already reported for the *Mimivirus* and *Megavirus chilensis* biosynthetic pathways for GlcN (Piacente *et al*. 2014a), Qui2N (Piacente *et al*. 2014b) and to those of *Campylobacter jejuni* for the biosynthesis of diNAcBac (Olivier and Imperiali 2008, Morrison and Imperiali 2014, Riegert *et al*. 2015, 2017). Sequence identity values with reference sequences are reported in Table 1. Structural multiple alignments were performed to assess conservation of the catalytic sites for each enzyme of the pathway and to infer whether *Moumouvirus australiensis* candidate enzymes could be functional.

**Figure 5.**
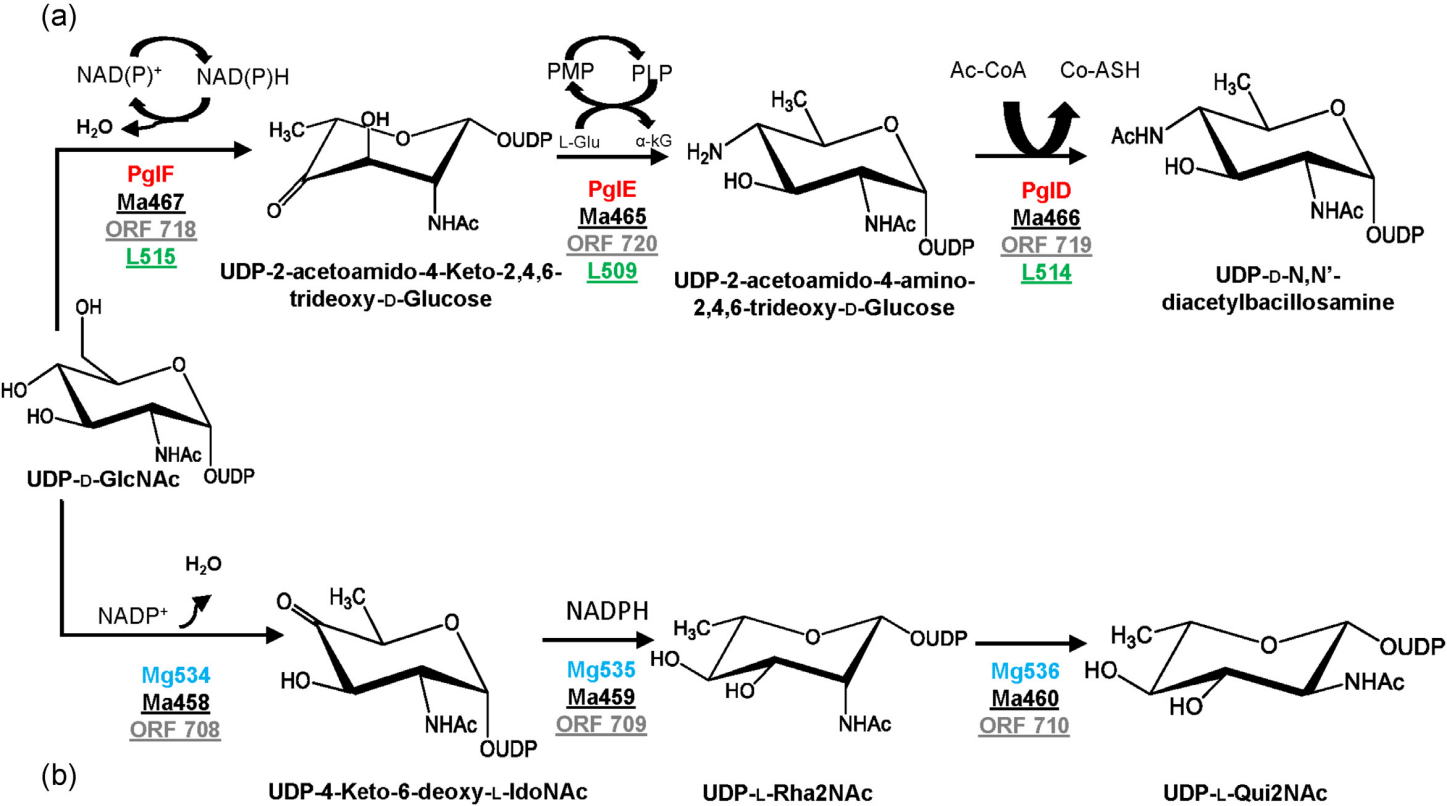
Proposed *Moumouvirus australiensis***(a)** UDP-d-diNAcBac pathway shared with *Tupanviruses* and **(b)** UDP-l-QuiNAc synthetic pathway shared with *Cotonvirus japonicus*. *Campylobacter jejuni* proteins (Pgl, red) are used as reference for the bacillosamine pathway and *Megavirus chilensis* proteins (Mg, blue) for the quinovosamine pathway. The corresponding *Moumouvirus australiensis* (Ma, black), *Cotonvirus japonicus* (ORF, dark gray) and *Tupanvirus* deep ocean (L, light green) proteins are underlined. The NCBI accession number of protein sequences, their lengths and functions are reported in Table 1.

**Table 1.** Identification of proteins responsible for nucleotide-sugars synthesis in *Moumouvirus australiensis* (B-clade). Based on known proteins involved in biosynthetis pathways for quinovosamine (UDP-l-Qui2N), bacillosamine (UDP-d-diNAcBac) and glucosamine (UDP-d-GlcNAc), we identified corresponding proteins in *Moumouvirus australiensis* and its orthologs in other viral genomes, such as *Tupanviruses* and *Cotonvirus japonicus*. For each pathway we reported proteins used as references, homologs in *Moumouvirus australiensis* and its closest orthologs in other viral genomes. For each protein, we show Accession number (NCBI database), % of identity (% Id.) and % of query coverage (% Cover).

	Reference protein			Ortholog in Moumouvirus australiensis				Orthologs in the proposed *Megavirinae* subfamily			
Pathway	Source	Name	Accession	Name	Accession	% Id.	%Cover	Source	Accession	% Id.	% Cover
**UDP-l-Qui2N**	*Megavirus chilensis*	Mg534	PDB: 4TQG	Ma458	AVL94844	79.50	99	*Cotonvirus*	BCS83197	78.26	99
								*Hyperionvirus*	AYV83503	61.84	93
								*KNV1*	ARF11838	57.01	99
								*CTV1*	ARF08716	57.85	99
		Mg535	YP_004894586	Ma459	AVL94845	59.48	99	*Cotonvirus*	BCS83198	65.19	99
								*KNV1*	ARF11657	48.91	98
								*CTV1*	ARF08710	45.59	99
		Mg536	YP_004894587	Ma460	AVL94846	70.00	99	*Cotonvirus*	BCS83199	72.78	100
								*KNV1*	ARF11840	60.38	98
**UDP-d-diNAcBac**	*Campylobacter jejuni*	PglF	PDB:5BJU	Ma467	AVL94853	27.95	79	*Moumouvirus*	YP_007354434	69.18	100
								*Moumouvirus*	YP_007354431	52.54	98
								*M. goulette*	AGF85279	67.38	100
								*M. monve*	AEX62730	52.54	98
								*Cotonvirus*	BCS83207	79.57	100
								*T. deep ocean*	QKU33921	78.49	100
								*T. soda lake*	QKU35168	78.49	100
		PglE	PDB:1O61	Ma465	AVL94851	33.19	60	*Cotonvirus*	BCS83209	75.32	99
								*T. deep ocean*	QKU33915	66.93	98
								*T. soda lake*	QKU35164	67.19	99
		PglD	PDB:3BSS	Ma466	AVL94852	30.69	95	*Cotonvirus*	BCS83208	68.78	98
								*T. deep ocean*	QKU33920	63.94	99
								*T. soda lake*	QKU35167	64.08	98
								*Mimivirus*	YP_003986634	45.19	99
**UDP-d-GlcNAc**	*Mimivirus*	L619	YP_003987136	Ma652	AVL95038	61.58	100	*Cotonvirus*	BCS83440	63.01	100
								*M. chilensis*	YP_004894796	58.62	100
								*T. deep ocean*	QKU33475	58.12	100
								*T. soda lake*	QKU34707	58.28	100
								*Hyperionvirus*	AYV83493	41.28	100
		L316	YP_003986819	Ma514	AVL94900	58.22	98	*Cotonvirus*	BCS83270	63.27	99
								*M. chilensis*	YP_004894641	56.16	98
								*T. deep ocean*	QKU33820	61.64	98
								*T. soda lake*	QKU35067	66.21	97
		R689	YP_003987216	Ma192	AVL94578	66.94	96	*Cotonvirus*	BCS82870	61	96
								*M. chilensis*	YP_004894291	65.59	96
								*T. deep ocean*	QKU34490	68.31	95
								*T. soda lake*	QKU35828	65.74	98

### UDP-d-N, N’-diacetyl-bacillosamine pathway (UDP-d-diNAcBac)

The UDP-d-diNAcBac pathway has been characterized in *Campylobacter jejuni* by determining catalytic activities and 3D structures of all its enzymes (Olivier and Imperiali 2008, Morrison and Imperiali 2014, Riegert *et al*. 2015, 2017). Synthesis of diNAcBac begins with GlcNAc and involves a three-step reaction: (1) dehydration (PglF), (2) transamination (PglE) and (3) acetylation (PglD). A Tblastn search using *C. jejuni* enzymes as queries (Table 2) enabled identification of Ma467, Ma465 and Ma466 as the first, second and third enzymes of the *Moumouvirus australiensis* pathway (Fig. 5a, Table 1).

**Table 2.** Identification of proteins responsible for nucleotide-sugars synthesis in *Moumouvirus maliensis* (B-clade). Based on known proteins involved in biosynthesis pathways for quinovosamine (UDP-d-Qui2N) and fucosamine (UDP-d-Fuc2N), we identified corresponding proteins in *Moumouvirus maliensis* and its closest orthologs in other giant virus genomes. For each pathway, we report proteins used as references, homologs in *Moumouvirus maliensis* and its orthologs in the proposed *Megavirinae* subfamily and in *Pseudomonas aeruginosa*. For each protein, we show Accession number (NCBI database), % of identity (% Id.) and % of query coverage (% Cover).

	Reference protein			Ortholog in Moumouvirus maliensis				Orthologs in the proposed *Megavirinae* subfamily				Orthologs in *Pseudomonas aeruginosa*		
Pathway	Source	Name	Accession	Name	Accession	% Id.	% Cover	Source	Accession	% Id.	% Cover	Accession	% Id.	% Cover
**UDP-d-Qui2Nor UDP-d_Fuc2N**	*C. jejuni*	PglF	PDB:5BJU	Mm422	QGR53991	30.05	59	*M. australiensis*	AVL94853	67,74	100	AAF72960	24,66	73
								*T. deep ocean*	QKU33921	68,46	100			
								*T. soda lake*	QKU35168	68,46	100			
	A. *thermoaerophilus*	Rmd	PDB:2PK3	Mm421	QGR53990	26,32	99	*T. deep ocean*	AUL79276	63,92	98	AAC45865	21,48	82
								*T. soda lake*	QKU35170	62,46	98	AF23991	22,85	98
				Mm419	QGR53988	31	29	*T. deep ocean*	QKU33921	53,99	98	AAC45865	19,5	52
								*T. soda lake*	QKU35168	53,99	98	AF23991	21,18	52
**UDP-d-GlcNAc**	*Mimivirus*	L619	YP_003987136	Mm595	QGR54170	61,25	100	*M. chilensis*	YP_004894796	58.62	100	—	—	—
								*T. deep ocean*	QKU33475	58.12	100			
								*T. soda lake*	QKU34707	58.28	100			
								*Hyperionvirus*	AYV83493	41.28	100			
		L316	YP_003986819	Mm467	QGR54037	57,53	98	*M. chiliensis*	YP_004894641	56.16	98	—	—	—
								*T. deep ocean*	QKU33820	61.64	98			
								*T. soda lake*	QKU35168	66.21	97			
		R689	YP_003987216	Mm152	QGR53719	67,35	96	*M. chiliensis*	YP_004894291	65.59	96	—	—	—
								*T. deep ocean*	QKU34490	68.31	95			
								*T. soda lake*	QKU35828	65.74	98			

Ma467 is conserved in all B-clade members (Table 1), while Ma465 and Ma466 have no homolog inside the B-clade. Surprisingly, their closest homologs are found in the *Tupanvirus* strains (D-clade) and *Cotonvirus japonicus* (E-clade) (Table 1). By contrast, lower sequence identities were found with the corresponding enzymes of *C. jejuni* (Table 1) (Riegert *et al*. 2017). Our *in silico* analyses of Ma467, Ma465 and Ma466 revealed that all catalytic residues are conserved (Figs. S3-S5), suggesting that these enzymes are functional and responsible for the biosynthesis of UDP-d-diNAcBac in *Moumouvirus australiensis* as suggested by the presence of diNAcBac in the fibrils of *Moumouvirus australiensis* (Fig. 3b). In addition, the identified biosynthetic pathway supports a d configured bacillosamine. Finally, in *Moumouvirus* gp464 and *Moumouvirus Monve* mvR525 proteins, which are homologues of Ma467, one of the catalytic aspartates is replaced by an asparagine (Fig. S3). This change has been associated with a loss of activity in *C. jejuni* D396N-PglF mutant (Riegert *et al*. 2017), suggesting that these strains (and the B-clade) could be in the process of losing the pathway.

### UDP-l-N-acetyl-quinovosamine pathway (UDP-l-Qui2NAc)

Previous studies had shown that two *Megavirus chilensis* proteins (Mg534, Mg535) were involved in UDP-l-Rha2NAc biosynthesis (Fig. 5b) and had predicted that a third protein (Mg536) could convert Rha2NAc into Qui2NAc (Piacente *et al*. 2014b). Although the function of Mg536 was not experimentally validated, the bioinformatic prediction is based on the identification of Qui2NAc as a component of the glycans decorating *Megavirus chilensis* fibrils (Fig. 3d).

Synthesis of Qui2NAc starts from GlcNAc and involves a five-step reaction and three enzymes (Fig 5b): (1 and 2) 4,6-dehydration and 5-epimerization (Mg534); (3 and 4) 4-reduction and 3-epimerization (Mg535); and (5) 2-epimerization (Mg536). Using these three proteins as queries, we identified corresponding orthologs in *Moumouvirus australiensis* (Ma458, Ma459 and Ma460) as the ones possibly involved in Qui2NAc production (Figs 5b and S6, Table 1). Ma458 corresponds to a 324-amino acid protein homologous to the *Megavirus chilensis* inverting UDP-GlcNAc 4,6-dehydratase, for which structure (PDB 4TQG) and activity were determined (Piacente *et al*. 2014b). Ma459 and Ma460 correspond respectively to 270- and 376-amino acid proteins with their closest homologues found in *Megavirus chilensis* (Mg535 and Mg536, respectively) (Table 1). Taken together, these results support the presence of a functional pathway for Qui2NAc in *Moumouvirus australiensis* (Fig. 5).

Ma458 is also present in *Cotonvirus japonicus* and in giant DNA viruses identified from metagenomics data (*Klosneuvirus* KNV1, *Catovirus* CTV1 and *Hyperionvirus*) (Schulz *et al*. 2017), while Ma459 is present in *Cotonvirus*, KNV1 and CTV1, and Ma460 is only present in *Cotonvirus* and in KNV1 (Table 1). Therefore, these results also support a functional pathway for Qui2NAc in *Cotonvirus* and KNV1 (Table 1). By contrast, CTV1 seems to only have the two first enzymes of the pathway, consequently restrained to Rha2N production.

### UDP-d-N-acetyl-glucosamine pathway (UDP-d-GlcNAc)

Although the amoeba host produces GlcNAc using typical eukaryotic enzymes, *Mimivirus* and *Megavirus chilensis* also possess necessary enzymes to synthesize this sugar (Piacente *et al*. 2014a) with intermediate properties between the eukaryotic and prokaryotic pathways. Using *Mimivirus* proteins (L619, L316 and R689) as queries, we identified corresponding enzymes in *Moumouvirus australiensis* (Ma652, Ma514 and Ma192, respectively; Table 1). Our *in silico* analyses suggested that the pathway for UDP-d-GlcNAc is conserved in members of all clades (Table 1) and that corresponding genes are scattered along the genome.

### Biosynthetic pathways for nucleotide-sugars in *Moumouvirus maliensis* (B-clade)

**Figure 6.**
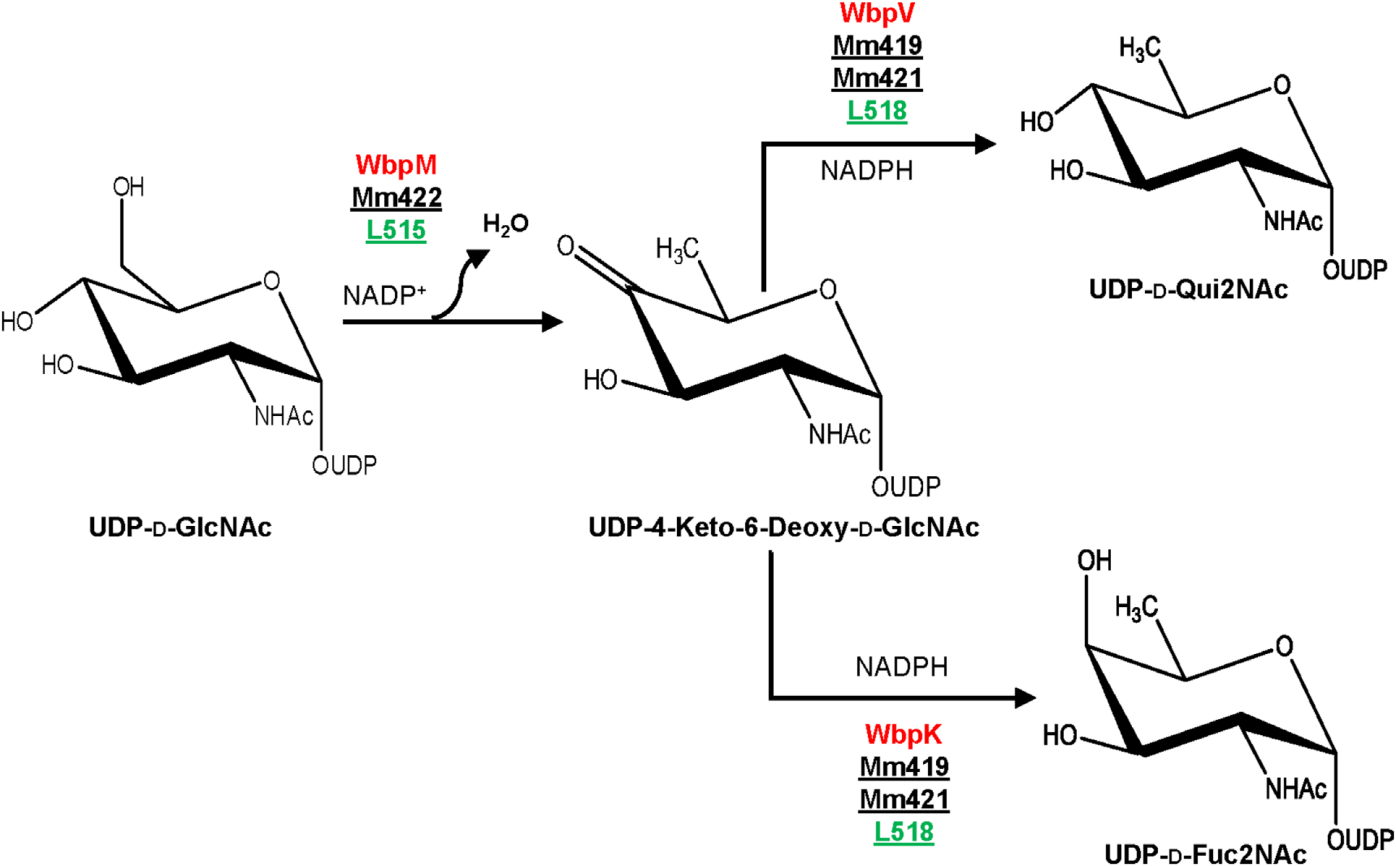
Proposed biosynthetic pathways for UDP-d-Qui2NAc and UDP-d-Fuc2NAc in B-clade (except *Moumouvirus australiensis*) and *Tupanviruses*. *Pseudomonas aeruginosa* reference proteins are in red and the corresponding *Moumouvirus maliensis* (black) and *Tupanvirus* deep ocean (light green) proteins are underlined. The NCBI accession number of the protein sequences, lengths and functions are reported in Table 2.

The chemical analysis of *Moumouvirus maliensis* virions revealed the presence of d-Fuc2N and d-Qui2N (Fig. 3c). Based on this finding, we searched for genes encoding proteins responsible for UDP-d-Qui2N and UDP-d-Fuc2N biosynthesis. Previous studies in bacteria showed that the precursor for both Qui2N and Fuc2N was UDP-d-GlcNAc (Burrows *et al*. 2000, Li *et al*. 2014) and that their synthesis involved a two-step reaction: (1) dehydration (UDP-GlcNAc 4,6 dehydratase), resulting in the intermediate UDP-4-keto-2-N-Acetyl-d-GlcNAc; and (2) reduction (4-reductase). Depending on the stereospecificity of the reductase, UDP-d-Qui2NAc or its C4-epimer UDP-d-Fuc2NAc are obtained (Fig. 6). Using bacterial enzymes as queries (Burrows *et al*. 2000, Li *et al*. 2014), we were able to identify corresponding enzymes in *Moumouvirus maliensis* (Fig. 6 and Table 2).

We found two proteins (Mm419, Mm422) homologous to *C. jejuni* 4,6-dehydratase (Table 2) (Riegert *et al*. 2017), but only Mm422 appeared to be functional, while Mm419 presented the mutation of the catalytic aspartate with asparagine (Fig. S7), which was reported to induce loss of activity (Riegert *et al*. 2017).

By using the *A*. *thermoaerophilus* 4-reductase (King *et al*. 2009) as a reference gene, we identified two putative 4-reductase enzymes (Mm419 and Mm421), which both have the typical consensus sequence for NADP binding and the catalytic triad common to all 4-reductase enzymes (Fig. S8, Table 2). At this stage, it is not possible to discriminate between the two 4-reductases to reach a complete understanding of the pathways for d-Fuc2N and d-Qui2N, as the stereospecificity of the two enzymes can only be assessed experimentally. In addition to the Fuc2N/Qui2N pathway, all the genes responsible for the GlcN production are also present and conserved compared with experimentally validated enzymes (Table 2). The low amount of GlcN revealed by our chemical analysis of *Moumouvirus maliensis* could possibly be due to conversion of most GlcN into Fuc2N and Qui2N.

### Biosynthetic pathways for nucleotide-sugars in *Cotonvirus japonicus* and *Tupanviruses*

In contrast to other clades (Fig. 3), the presence of sugars associated with the fibrils of *Cotonvirus japonicus* and *Tupanviruses* has not been experimentally characterized. Here, we searched their genomes for genes encoding for possible biosynthetic pathways for nucleotide-sugars. We used protein sequences of enzymes involved in the pathways identified for the A- (Piacente *et al*. 2012, 2014a, 2017b), B- (Tables 1 and 2) and C-clades (Piacente *et al*. 2014b) as queries. We found that *Cotonvirus japonicus* encodes a pathway for UDP-l-Qui2NAc shared with *Megavirus chilensis* and *Moumouvirus australiensis* (Figs. 5b and S6, Table 1). Both *Cotonvirus japonicus* and *Tupanvirus* strains share the biosynthetic pathway for UDP-d-diNAcBac with *Moumouvirus australiensis* (Table 1, Fig. 5a), with conservation of all catalytic residues suggesting all enzymes can be functional (Figs S3, S4 and S5). In addition, *Tupanviruses* possess the biosynthetic pathway for UDP-d-Qui2N/Fuc2N (Figs 6 and S7, Table 2) conserved in members of the B-clade, except in *Moumouvirus australiensis*. The first enzyme of the pathway (L515) is also the first enzyme in the diNAcBac pathway and is conserved in *Moumouvirus australiensis* (Fig. S7). However, in contrast to *Moumouvirus australiensis*, *Tupanviruses* possess the second enzyme (L518) corresponding to the 4-reductase in *Moumouvirus maliensis* (Mm421). The catalytic triad of this enzyme is conserved (Table 2, Fig. S8), suggesting that the entire pathway should be functional in *Tupanviruses*.

In the vicinity of the genes encoding the diNAcBac pathway in *Tupanviruses*, we identified the *R520* gene as encoding an UDP-glucose-6-dehydrogenase enzyme. This result suggests that *Tupanvirus* could convert UDP-d-glucose (UDP-d-Glc) into UDP-d-glucuronic acid (UDP-d-GlcA) (Fig. S9a). The closest homologs of R520 are found in KNV1 and *Cafeteria roenbergensis* virus (CroV), which also belongs to the *Mimiviridae* family (Fischer *et al*. 2010). Using HHpred for remote homology and structure prediction (Hildebrand *et al*. 2009), we found that *Burkholderia cepacia* UDP-glucose-6-dehydrogenase (PDB:2Y0C) was homologous to R520 (30% of identity and 100% confidence). Comparison of their sequences revealed that all catalytic residues were conserved in R520, suggesting that it is a functional enzyme (Fig. S9). In contrast to the other clades that use UDP-d-Glc from their host, *Tupanviruses* possess the *L502* gene encoding a 634-amino acid protein with a predicted glucose-1P-uridyltransferase N-terminal domain (confidence 99%). This enzyme uses glucose-1P to synthesize UDP-d-Glc, allowing *Tupanviruses* to be completely independent from the host for glycosylation. The biosynthetic pathway for UDP-d-Glc is present in the amoeba host and is expressed during the late stage of the infection, as is seen for the viral pathway. Therefore, we cannot exclude that viruses could also recruit the host UDP-d-Glc to build their own glycans.

### Genomic organization of glyco-related genes in the different clades

**Figure 7.**
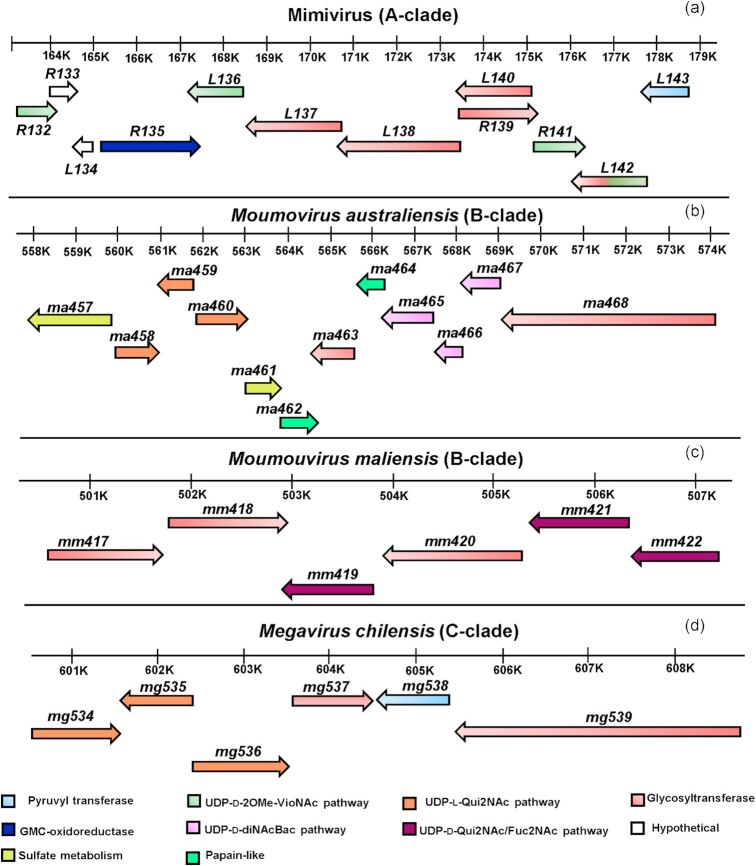
Organization of the glycosylation gene clusters of **(a)***Mimivirus*, involved in the biosynthesis of two polysaccharides (Notaro *et al*. 2021) and used as prototype for A-clade; **(b)** for B-clade outsider *Moumouvirus australiensis* and **(c)** for the B-clade prototype *Moumouvirus maliensis*; **(d)** for C-clade prototype *Megavirus chilensis*. The arrows' direction indicates the coding strand. The function of each gene is color-coded (explained in the legend) and reported in Table 3.

**Figure 8.**
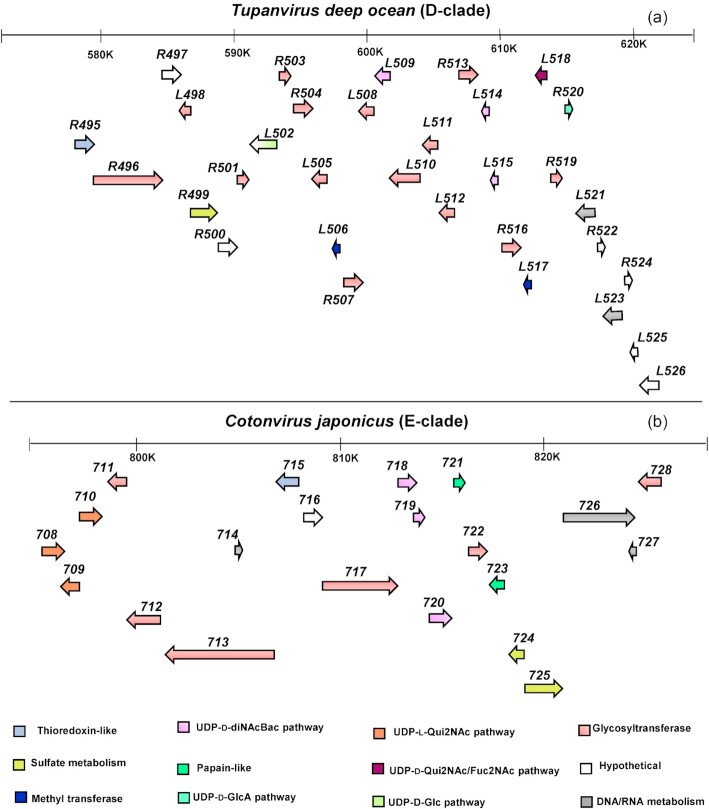
Organization of the glycosylation gene clusters of **(a)***Tupanvirus deep ocean* as prototype of the two Tupanviruses strains. **(b)***Cotonvirus japonicus* as prototype of a new clade isolated in 2021. The arrows' direction indicates the coding strand. The function of each gene is color-coded (explained in the legend) and it is reported in Tables 4 and 5.

Our analysis of the genomes of prototypical viruses from different clades revealed that most genes responsible for the synthesis of nucleotide-sugars, along with others likely involved in the glycosylation process, are located within the same region of the genome, namely in gene clusters (Figs. 7 and 8, Tables 3, 4 and 5).

**Table 3.** Glycosylation gene clusters for *Mimivirus*, *Moumouvirus australiensis*, *Moumouvirus maliensis* and *Megavirus chilensis*. For each virus, the NCBI Accession number of the complete genome and the genes names in each cluster are reported as well as the Accession number of the encoded protein, its length and expected function.

Source	Genome accession #	Gene	Protein accession #	Length (aa)	Predicted function
*Mimivirus* (A-clade)	NC_014649	*R132*	YP_003 986 624	221	Methyltransferase
		*R133*	YP_003 986 625	127	Hypothetical
		*L134*	YP_003 986 626	163	Hypothetical
		*R135*	YP_003 986 627	702	GMC-type oxidoreductase
		*L136*	YP_003 986 628	352	Pyridoxal phosphate-dependent transferase
		*L137*	YP_003 986 629	732	Glycosyltransferase
		*L138*	YP_003 986 630	883	Glycosyltransferase
		*R139*	YP_003 986 631	248	Glycosyltransferase
		*L140*	YP_003 986 632	304	Glycosyltransferase
		*R141*	YP_003 986 633	323	4,6-dehydratase
		*L142*	YP_003 986 634	490	N-ter:acetyltransferase, C-ter: glycosyltransferase
		*L143*	YP_003 986 635	287	Piruvyltransferase
*Moumouvirus australiensis* (B-clade)	MG807320	*ma457*	AVL94843	642	Adenylyl-sulfate kinase
		*ma458*	AVL94844	324	4,6-dehydratase, 5-epimerase
		*ma459*	AVL94845	270	4-reductase
		*ma460*	AVL94846	376	2-epimerase
		*ma461*	AVL94847	263	Sulfotransferase*
		*ma462*	AVL94848	188	Papain-like*
		*ma463*	AVL94849	325	Glycosyltransferase*
		*ma464*	AVL94850	204	Papain-like*
		*ma465*	AVL94851	384	Aminotransferase
		*ma466*	AVL94852	209	Acetyltransferase
		*ma467*	AVL94853	278	4,6-dehydratase
		*ma468*	AVL94854	1666	Glycosyltransferase
*Moumouvirus maliensis* (B-clade)	MK978772	*mm417*	QGR53986	389	Glycosyltransferase (GT-B)
		*mm418*	QGR53987	386	Glycosyltransferase (GT-B)
		*mm419*	QGR53988	278	4-reductase
		*mm420*	QGR53989	467	Glycosyltransferase
		*mm421*	QGR53990	321	4-reductase
		*mm422*	QGR53991	279	4,6-dehydratase
*Megavirus chilensis* (C-clade)	NC_016 072	*mg534*	PDB: 4TQG	323	4,6-dehydratase, 5-epimerase
		*mg535*	YP_004 894 586	270	4-reductase
		*mg536*	YP_004 894 587	370	2-epimerase
		*mg537*	YP_004 894 588	312	Putative GT*
		*mg538*	YP_004 894 589	277	Piruvyltransferase
		*mg539*	YP_004 894 590	1097	Glycosyltransferase

* indicates that the protein function was predicted with >99% confidence by HHpred.

**Table 5.** Glycosylation gene cluster for *Cotonvirus japonicus*. The NCBI Accession number of the complete genome and the name of the genes in the cluster are reported as well as the Accession number of the encoded protein, its length and expected function.

Genome accession #	Gene	Protein accession #	Length (aa)	Predicted function
AP024483	*ORF_708*	BCS83197	324	4,6-dehydratase, 5-epimerase
	*ORF_709*	BCS83198	271	4-reductase
	*ORF_710*	BCS83199	372	2-epimerase
	*ORF_711*	BCS83200	285	Glycosyltransferase
	*ORF_712*	BCS83201	564	Glycosyltransferase*
	*ORF_713*	BCS83202	1802	Glycosyltransferase
	*ORF_714*	BCS83203	138	ABC transporter permease protein YphD
	*ORF_715*	BCS83204	361	Thioredoxin-like
	*ORF_716*	BCS83205	286	Hypothetical
	*ORF_717*	BCS83206	1196	Glycosyltransferase
	*ORF_718*	BCS83207	279	4-epimerase
	*ORF_719*	BCS83208	210	Acetyltransferase
	*ORF_720*	BCS83209	386	Aminotransferase
	*ORF_721*	BCS83210	208	Papain-like
	*ORF_722*	BCS83211	336	Glycosyltransferase*
	*ORF_723*	BCS83212	265	Papain-like
	*ORF_724*	BCS83213	264	Sulfotransferase
	*ORF_725*	BCS83214	624	Adenylyl-sulfate kinase
	*ORF_726*	BCS83215	1092	Muts-like protein
	*ORF_727*	BCS83216	73	DNA-directed RNA polymerase
	*ORF_728*	BCS83217	371	Glycosyltransferase*

* indicates that the protein function was predicted with >99% confidence by HHpred.

**Table 4.** Glycosylation gene clusters for *Tupanviruses*. The NCBI Accession number of the complete genomes and the names of the genes in each cluster are reported as well as the Accession number of the encoded protein, its length and expected function.

Tupanvirus deep ocean					Tupanvirus soda lake		
Genome accession #	Gene	Protein accession #	Length (aa)	Predicted function	Protein accession	%ID	%Cover
MF405918	R496	QKU33902	1707	Glycosyltransferase	QKU35148	83.63	100
	R497	QKU33903	475	Hypothetical	QKU35149.	85.09	96
	L498	QKU33904	266	Glycosyltransferase*	QKU35150	82.17	86
	R499	QKU33905	655	Adenylyl-sulfate kinase	QKU35151	88.85	100
	R500	QKU33906	453	Hypothetical	QKU35152	80.49	99
	R501	QKU33907	291	Glycosyltransferase*	QKU35153	80.80	94
	L502	QKU33908	639	Glycosyltransferase	QKU35154	90.27	99
	R503	QKU33909	334	Glycosyltransferase*	QKU35155.	84.73	100
	R504	QKU33910	510	Glycosyltransferase*	QKU35156	83.53	100
	L505	QKU33911	365	Glycosyltransferase*	QKU35157	74.52	100
	L506	QKU33912	219	Methyltransferase*	QKU35158	84.72	98
	R507	QKU33913	425	Glycosyltransferase*	QKU35159	80.19	99
	L508	QKU33914	373	Glycosyltransferase*	QKU35161	61.20	97
	L509	QKU33915	388	Aminotransferase	QKU35164.	87.99	98
	L510	QKU33916	761	Multi GT domains*	QKU35165	57.49	93
	L511	QKU33917	367	Multi GT domains	QKU35162	73.00	100
	L512	QKU33918	448	Glycosyltransferase*	QKU35165	55.19	81
	R513	QKU33919	509	Glycosyltransferase*	QKU35166	59.49	100
	L514	QKU33920	209	Acetyltransferase	QKU35167	79.13	98
	L515	QKU33921	279	4-epimerase	QKU35168	87.81	100
	R516	QKU33922	546	Glycosyltransferase*	QKU35169	63.08	83
	L517	QKU33923	194	Methyltransferase*	—	—	—
	L518	AUL79276	321	4-reductase	QKU35170	90.88	99
	R519	QKU33924	360	Glycosyltransferase	QKU35171	87.53	100
	R520	QKU33925	268	UDP-glucose 6-dehydrogenase	QKU35172	91.76	95
	L521	QKU33926	506	Bifunctional glutamate/proline tRNA-synthetase	QKU35173	86.17	100
	R522	QKU33927	172	Hypothetical	AUL77993	43.20	94
	L523	QKU33928	445	Histidine tRNA synthetase	QKU35174	85.20	100
	R524	QKU33929	188	Hypothetical	QKU35176	85.11	100
	L525	AUL79282	200	Hypothetical	QKU35177	81.59	100
	L526	QKU33930	511	Hypothetical	QKU35178	62.44	66

* indicates that the protein function was predicted with >99% confidence by HHpred.

For *Mimivirus* (A-clade), we identified *R132*, which encodes a Vio4NAc methyltransferase (Fig. 4), and expanded the original cluster from nine (Piacente *et al*. 2012) to 12 genes (Fig. 7a, Table 3). This larger cluster also includes *R133* and *L134*, which encode two ORFans proteins whose function in glycosylation remains to be determined (Fig. 7a, Table 3).

For *Moumouvirus australiensis* (B-clade), we identified gene clusters responsible for l-Qui2NAc (*ma458*, *ma459*, *ma460*) and d-diNAcBac (*ma465*, *ma466*, *ma467*) synthesis. Next to these genes, we found others that are also related to glycosylation, allowing us to define a larger cluster containing 12 genes (Fig. 7b, Table 3). Two of these genes encode proteins involved in sulfate metabolism (*ma457*, *ma461*), with Ma457 predicted as a bifunctional Sulfate adenylyltransferase/Adenosine-5'-phosphosulfate kinase and Ma461 as a sulfotransferase. This result suggests that sugars could be further modified by a sulfate group, but experimental validation is needed. In the same region, we found two predicted papain-like proteins (Ma462 and Ma464) that are not strictly related to glycosylation and a predicted glycosyltransferase (Ma463), which is related to glycosyltransferases (GTs) from *Streptococcus sanguinis* (5V4A) and *S. parasanguinis* (4PHR). In addition, *ma468* encodes a 1666-amino acids protein with four GT domains. *Moumouvirus australiensis* thus contains the genes necessary for its nucleotide-sugars production as well as the GTs responsible for their assembly into oligosaccharides or polysaccharides.

For *Moumouvirus maliensis* (B-clade), we identified a smaller six-gene cluster (Fig. 7c, Table 3) that includes genes for UDP-d-Qui2NAc and UDP-d-Fuc2NAc biosynthesis, and three genes (*mm417*, *mm418* and *mm420*) encoding putative GT enzymes.

For *Megavirus chilensis* (C-clade), we had also identified a six-gene cluster (Piacente *et al*. 2014b), which encodes proteins involved in Rha2NAc (Mg534, Mg535) and l-Qui2NAc (Mg534, Mg535, Mg536) production, as well as a protein with three GT domains (Mg539), a hypothetical protein (Mg537) and a pyruvyltransferase (Mg538) which could play the same role as *Mimivirus* L143 (Fig. 7d, Table 3). We found that the closest homologs of Mg537 are *Streptococcus* GTs (PDB codes: 4PHR and 5V4A, 99% confidence), also identified above for Ma463. These results suggest that Mg537 and Mg539 could correspond to GTs involved in the C-clade glycosylation pathway.

In accordance with the complexity of their decorated capsids and tails, *Tupanviruses* (D-clade) also have a bigger and more complex glycosylation gene cluster covering a region of 49 Kb with up to 33 genes (Fig. 8a, Table 4). This cluster includes several biosynthetic pathways for nucleotide-sugars such as UDP-d-diNAcBac (*L515*, *L509* and *L514*), UDP-d-Qui2N/Fuc2N (*L515* and *L518*) and UDP-d-glucuronic acid (*R520*), suggesting that fibrils decorating the capsids and tails may also be glycosylated. Their sugars could be further modified by sugar methyltransferases (L506 and L517) present in the cluster (Fig. 8a). We also found a SAT/APS kinase homolog of Ma457, but the homolog of the sulfotransferase (Ma461) is missing. As a result, experimental validation of these modifications is needed. Compared with other clades (Fig. 7), *Tupanviruses* present an increased number of GTs (Fig. 8, Table 4), with 11 genes encoding a single GT domain and two genes (*R496*, *L510*) encoding four and two GT domains, respectively. Final glycan structures could be very complex and heterogeneous reflecting the presence of fibrils decorating both capsids and tails. Two additional enzymes (L521 and L523) clearly related to RNA metabolism (see Table 4 for details) were identified in the cluster as well as ORFans proteins (R497, R500, R522, R524, L525 and L526). Their function remains to be determined.

For *Cotonvirus japonicus* (E-clade), biosynthetic pathways for UDP-l-Qui2NAc (ORFs *708*, *709* and *710)* and UDP-d-diNAcBac (ORFs *718*, *719* and *720*) are conserved and next to other glycogenes. This allowed us to define a 21-gene cluster in *Cotonvirus* (Fig. 8b, Table 5). This cluster includes seven GTs with some homology to *Moumouvirus australiensis* GTs. For instance, GT 713 and 717 share 58 and 40% identities with Ma468 with a coverage of 70 and 95%, respectively, while GT 722 and 712 share 72 and 29% identity with Ma463 on the entire protein sequences. In addition, two enzymes involved in the sulfate metabolism were identified as ORFs 724 and 725 that correspond to Ma461 and Ma457 enzymes, with identities up to 60% along the entire protein sequences (Table 5). Inside the cluster, there are genes not related to glycosylation such as two predicted papain-like proteins (ORFs *721* and *723*), a permease, MutS and an RNA polymerase subunit (ORFs *714*, *726* and *727*) (Table 5).

Except for *Mimivirus*, gene clusters involved in proposed *Megavirinae* glycosylation are located between a conserved helicase and a thioredoxin-like gene (Fig. S10). The number of genes in this region ranges from six genes for B- and C-clades to 12 genes for *Moumouvirus australiensis* and up to 33 genes for *Tupanviruses*. In *Mimivirus*, this region is only 3.5 kb long and only includes one GT (R363; Fig. S10), while the 12-gene cluster (Fig. 7a) is located in a different genomic region between a putative transcription factor gene (*R131*) and an orfan gene (*L144*). In *Cotonvirus japonicus*, only seven glycogenes of the glycosylation gene cluster (Fig. 8b) lie between the helicase and thioredoxin-like genes (Fig. S10). This part of the cluster presents strong homology with the *Megavirus chilensis* cluster and includes the l-Qui2N pathway and one GT. After the thioredoxin-like gene (*715*), there are the remaining 14 glycogenes that are part of the 21-gene cluster (Figs 8b and S10).

### Expanding the glycosylation feature of each prototype to the clade

**Figure 9.**
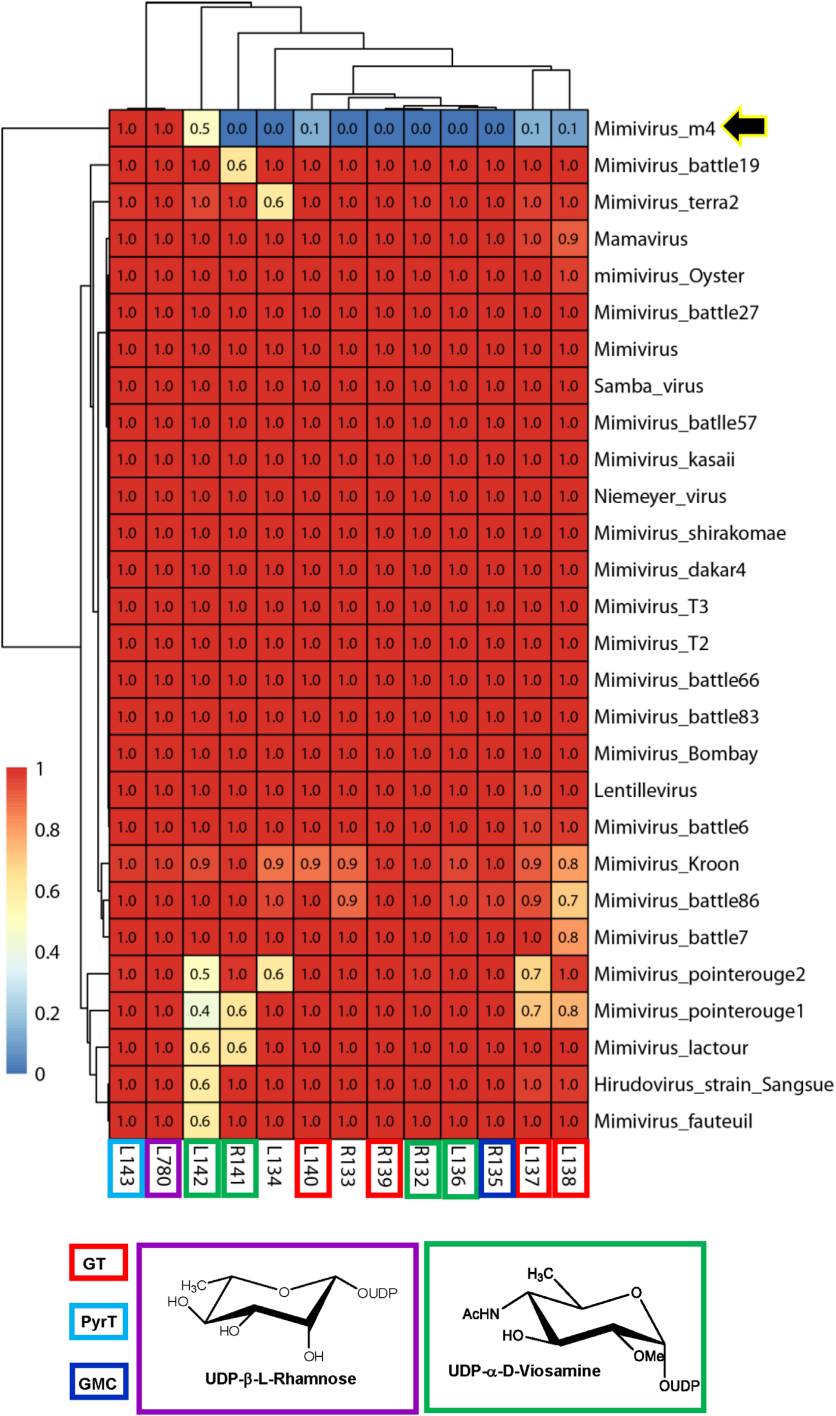
Heatmap based on the level of conservation of the *Mimivirus* 12-glycogenes cluster in the A-clade. The reference *Mimivirus* L780 (in violet) and R141 responsible for Rha production have also been included. Proteins involved in UDP-d-2OMeVio4NAc biosynthesis are marked in green; the pyruvyltransferase (L143) involved in modification of GlcNAc in poly_1 of *Mimivirus* (Fig. 2) is shown in light blue; all glycosyltransferases (GTs) are labeled in red. R135 (dark blue) is the GMC-oxidoreductase composing the fibrils. R133 and L134 are ORFans. The black arrow indicates M4, which lacks the gene cluster. The conservation score for each protein in the different genomes ranges from 0 to 1 (low to high: blue to red).

**Figure 10.**
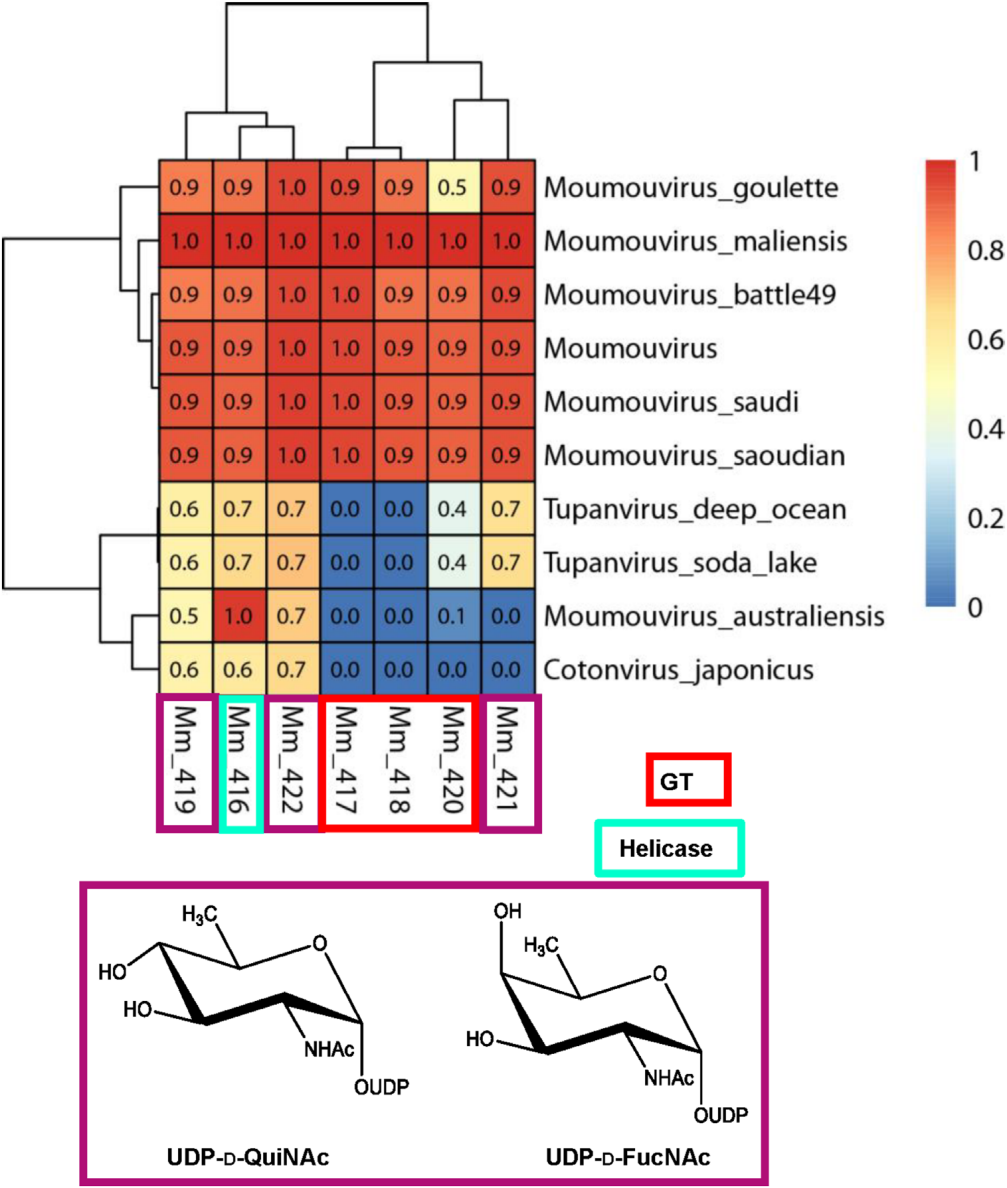
Heatmap of the conservation level of the *Moumouvirus maliensis* 6-glycogene cluster in the B-clade, D-clade (*Tupanviruses*) and E-clade (*Cotonvirus japonicus)*. Proteins involved in UDP-d-Qui2NAc/FucNAc biosynthesis are marked in violet, while glycosyltransferases (GTs) are in red. The helicase Mm416 (light green) is used for reference of the conservation level between clades. The conservation score for each protein in the different genomes ranges from 0 to 1 (low to high: blue to red).

**Figure 11.**
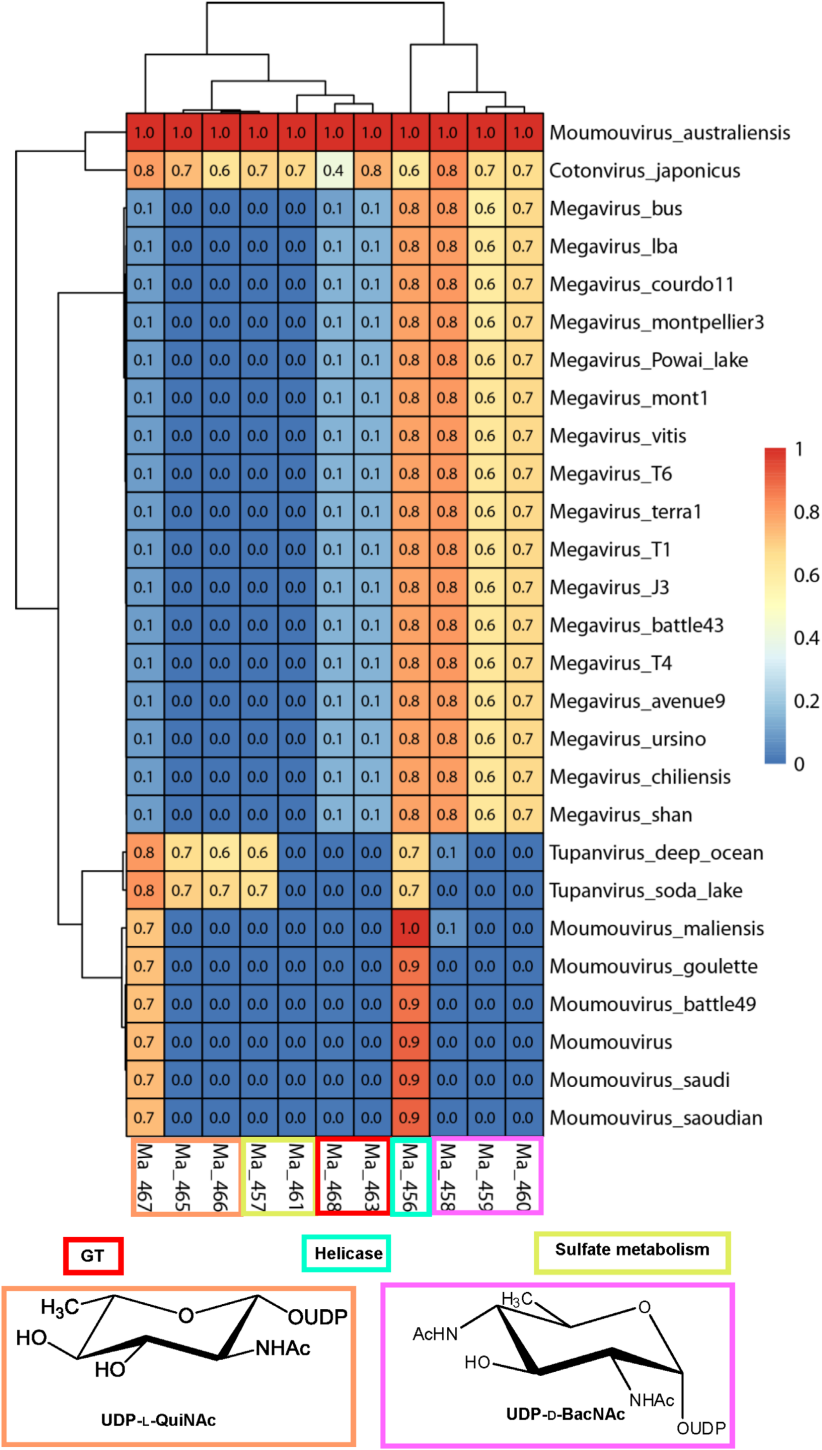
Heatmap of the conservation level of the *Moumouvirus australiensis* 12-glycogene cluster in the B-clade, C-clade, D-clade (*Tupanviruses*) and E-clade (*Cotonvirus japonicus)*. Proteins involved in the UDP-l-Qui2NAc and UDP-d-BacNAc biosynthetic pathways are in orange and pink, respectively. The GTs are in red, while proteins responsible for sulfate metabolism are in dark yellow. The helicase Ma456 (in cyan) is used for reference of the conservation level between clades. The two papain-like proteins (Ma462, Ma464) are not included because they are not directly related to fibrils glycosylation. The conservation score for each protein in the different genomes ranges from 0 to 1 (low to high: blue to red).

To gain further insights into the evolution of the glycosylation machinery in the proposed *Megavirinae* subfamily, we examined conservation of the gene clusters (Figs 7 and 8) within and among clades. We only used clade members for which the genome sequence was complete (Figs 9, 10, 11 and S11;,Table S1).

For the A-clade, our analysis shows that enzymes for UDP-l-Rha (R141, L780) and UDP-d-2OMe-Vio4NAc (R132, L136, R141 and L142) biosynthesis are conserved (Fig. 9). While L780 (YP_003 987 312; 289 aa) does not belong to the gene cluster (Fig. 7a), it was included because it is the second enzyme in the Rha biosynthesis pathway (Parakkottil Chothi *et al*. 2010). The highest divergence was obtained for L142 (Table 3), which is a bifunctional enzyme with an N-terminal N-acetyltransferase domain responsible for Viosamine acetylation and a C-terminal putative GT domain, which could transfer Vio4NAc onto its acceptor (Piacente *et al*. 2017a). This divergence can be explained by the split (or fusion) of the *L142* gene leading to two genes in Mimivirus M4 (*L142a* and *L142b*) and in *Hirudovirus* strain *sangsue* (*HIUR_S825* and *HIUR_S826*), where the first ones correspond to the N-acetyltransferase domain and the second ones to the GT domain. Moreover, all GTs in the *Mimivirus* gene cluster (L137, L138, L139, R140 and the C-ter L142), predicted as type 2 GTs in the Carbohydrate-Active enZYmes database (CAZY) (Cantarel *et al*. 2009), are specific to the A-clade and are likely involved in building the two polysaccharides constituting Mimivirus fibrils (Fig. 2) (Notaro *et al*. 2021). They share a high level of sequence identity (Table S3), as expected because the repeated units of the two glycans include Rha and GlcN linked in a different way (Fig. 2) (Notaro *et al*. 2021). R133 and L134 are hypothetical proteins that seem to be specific of the A-clade and at this stage it is not possible to conclude on their possible contribution to fibril glycosylation or maturation. Inside the A-clade, the laboratory strain *Mimivirus* M4, which lacks fibrils (Boyer *et al*. 2011a), is the only one without a glycosylation cluster (Fig. 9, yellow arrow) and without the proteins constituting *Mimivirus* fibrils.

In line with the A-clade, the *Moumouvirus maliensis* six-gene cluster is conserved in B-clade except for *Moumouvirus australiensis* (Fig. 10).

*Moumouvirus australiensis*, *Cotonvirus japonicus* and the two *Tupanvirus* strains share with the B-clade the 4,6-dehydratase enzyme (Mm422) involved both in diNAcBac and Qui2N/Fuc2N production (Fig. 11) as well as the non-functional Mm419 protein.

*Tupanviruses* share the UDP-d-Qui2NAc/Fuc2NAc biosynthetic pathway with the B-clade, as evidenced by the presence of a 4-reductase enzyme homolog to *Moumouvirus maliensis* Mm421, as well as a glycosyltransferase homolog to Mm420. Despite clearly belonging to the B-clade phylogenetic tree when using conserved core genes (Fig. 1), *Moumouvirus australiensis* appears as an outsider of the B-clade for its glycosylation genes (Figures 10 and 11). Enzymes involved in UDP-l-Qui2NAc biosynthesis (Ma458, Ma459 and Ma460) and glycosyltransferases (Ma463 and Ma468) are completely absent from the B-clade.

Even more surprisingly in terms of evolution, *Moumouvirus australiensis* shares the UDP-l-Qui2N biosynthesis pathway with the entire C-clade and *Cotonvirus japonicus* (Fig. 11), while it shares the UDP-d-diNAcBac biosynthesis pathway with *Cotonvirus japonicus* and the more distant *Tupanvirus* strains (Fig. 11). Enzymes involved in sulfate modification of sugars (Ma457 and Ma461) are both conserved in *Cotonvirus japonicus*. By contrast, only one enzyme remains in *Tupanviruses* (Ma457) (Fig. 11). The *Moumouvirus australiensis* gene cluster is completely conserved in *Cotonvirus japonicus* (Fig. 11), but the gene cluster of *Cotonvirus* counts 21 genes instead of 12 and includes several additional glycosyltransferases and hypothetical proteins (Fig. 8, Table 4). These data allowed us to address the evolutionary position of *Moumouvirus australiensis* between B-clade, *Cotonvirus japonicus* and *Tupanviruses* with whom it appears closely related in terms of glycosylation (Fig. 11).

For the C-clade, all enzymes involved in UDP-l-RhaNAc (Mg534, Mg 535) and Qui2NAc synthesis (Mg534, Mg535 and Mg536 homologues of Ma458, Ma459 and Ma460) are conserved inside the clade. In addition, the two GTs (Mg537, Mg539) are also conserved and distant from the *Moumouvirus australiensis* ones (Ma463, Ma468) (Fig. 11).

This analysis confirmed our initial hypothesis that the glycosylation machinery is clade-specific and suggests that *Moumouvirus australiensis* likely represents an intermediate prototype for the evolution of a new glycosylation cassette.

Finally, the UDP-d-GlcNAc biosynthesis pathway is conserved in the five clades and the corresponding genes are not arranged into clusters (Fig. S11). In addition, the A-clade, C-clade and *Cotonvirus japonicus* share a putative pyruvyltransferase (Fig. S11) that could be involved in GlcNAc modification with pyruvic acid, as is observed in *Mimivirus* polysaccharides (Fig. 2) (Notaro *et al*. 2021). Even if GlcNAc is present in the amoeba host, all giant DNA viruses encode their own proteins to synthesize this sugar, which is also the precursor of other 6-deoxy-amino-sugars (Rha2NAc, Qui2NAc, diNacBac and Fuc2NAc) that constitute the capsid fibrils of the different members of the three clades.

## Discussion

Here, we show that complex gene clusters are involved in glycosylation of the fibril layer surrounding the viral particles in the proposed *Megavirinae* subfamily (Figs 7 and 8). However, fibril glycosylation occurs in a clade-specific manner, suggesting a high degree of variability in glycan structures for the different members of the family. Our analyses raise two important questions: (i) What are the possible evolutionary implications of organizing glycosylation genes into clusters? and (ii) What is the role played by sugars constituting the fibrils for the different clades? Answering these questions is essential for a clear understanding of glycosylation in giant viruses.

### Implications of clustering glycogenes

Organization of glycogenes into clusters is reminiscent of what occurs in bacteria. For example, genes involved in biosynthesis of lipopolysaccharide (LPS), the main component of the Gram-negative bacteria outer membrane, are organized in a cluster controlled by a unique promoter defining the *waa* operon in *Escherichia coli* K12 (Gronow and Brade 2001). Similarly, genes responsible for the biosynthesis of the N-/O-glycans that decorate flagellin or pili are organized into operons (all controlled by the same promoter). *Campylobacter jejuni* presents a cluster of genes responsible for flagellin glycosylation, called pgl, which is further organized in two operons: operon I includes *pglB*, *pglA*, *pglC* and *pglD*, while operon II contains *pglE*, *pglF* and *pglG* (Szymanski *et al*. 1999). Interestingly, *pglE* and *pglF* are co-transcribed, while *pglD* transcription is regulated by another operon system (Szymanski *et al*. 1999), despite all being involved in UDP-d-diNAcBAc biosynthesis. Because operons are often controlled by a single promoter, levels and timing of expression are comparable for all the genes.

It is currently unknown how transcription and expression of glycogenes occur in giant viruses. The transcriptomes of *Mimivirus* (Legendre *et al*. 2010) and *Megavirus chilensis* (Arslan *et al*. 2011) being the only ones available, they are used as reference for the entire family. We had previously showed that there are three main expression classes: early (from T = 0 to T = 3 h),  intermediate (from T = 3 h to T = 6 h) and late (from T = 6 h to T = 12 h). Only early and late promoters have been identified, with a highly stringent early promoter (AAAATTGA) (Suhre *et al*. 2005) and a less stringent late promoter (Legendre *et al*. 2010). All glycogenes are expressed in the late stage of the infection cycle whether they are organized into a cluster or not. *Mimivirus* glycosylation genes are clustered (Fig. 7a) and all have their own late promoter, except *R133*, which encodes a hypothetical protein. All are expressed in the late phase (6–12 hours post infection ) of the infectious cycle, supporting a role for this cluster in fibril glycosylation. On the contrary, for genes in the *Megavirus chilensis* glycosylation cluster (Fig. 7d), only *mg535*, *mg536* and *mg539* have a promoter. Given the low conservation of the late promoter, it could have been missed by the annotation or these genes are expressed as polycistronic mRNAs, as shown previously for some *Mimivirus* genes (Byrne *et al*. 2009).

Previous studies (Piacente *et al*. 2012, 2014a, 2015) and this one show that it is highly challenging to establish the origin of these glycosylation gene clusters. For the viosamine synthesis pathway, the patchwork of bacterial-like and eukaryotic-like genes composing the cluster precludes the hypothesis that they were all acquired at once from a single organism (Piacente *et al*. 2012). To detect putative horizontal gene transfer, we compared the GC content of glycogenes (Figs 7 and 8) with *Mimiviridae* core genes (Fig. S12). We showed that glycogenes are as AT-rich as the overall genome, suggesting that they could have been acquired early in evolution so that their GC content had time to evolve. Giant viruses possess up to five genes encoding transposases that might catalyze integrations of foreign DNA into their genomes, but these genes are far (more than 30 Kb) from the genomic region containing the glycogenes. In addition, the fact that glycogenes have the typical viral promoter (Legendre *et al*. 2010) could also suggest that giant viruses have evolved their own glycosylation machinery prior to the radiation of eukaryotes.

We showed that glycosylation gene-clusters of the B-clade (*Moumouviruses*), C-clade (*Megaviruses*), D-clade (*Tupanviruses*) and E-clade (*Cotonvirus japonicus*) are all located between conserved helicase and thioredoxin-like genes (Fig. S10). This result suggests that giant viruses could have exchanged these glycogenes through homologous recombination events or horizontal gene transfer inside the amoeba host, by analogy with bacteria (Aydanian *et al*. 2011). However, further studies are required to experimentally prove that these genes can be exchanged between giant viruses belonging to different clades and eventually with bacteria.

### Bacteria-like sugars as markers of proposed *Megavirinae* clades

From a structural point of view, the type of sugars found in giant viruses is drastically different from what was reported for eukaryotic viruses. For example, SARS-Cov-2 virus exhibits oligosaccharides of discrete size made by sugar units typical of the eukaryotic world, such as glucosamine, galactosamine, fucose and sialic acid (Zhao *et al*. 2021). By contrast, members of the proposed *Megavirinae* subfamily possess their own glycosylation machinery to synthesize and decorate their fibrils with rare amino sugars that are only encountered in bacteria and are absent from their amoeba host. The organization of these glycogenes into clusters also increases the evolvability of the glycosylation machinery resulting in different sugars between clades and even inside the same clade, as was observed for *Moumouvirus australiensis*.

By comparing the conservation level of glyco-enzymes, we identified one or two sugars as markers of a specific clade. A-clade is characterized by the rare sugar d-viosamine, which is a component of *Pseudomonas syringe* flagellin (Yamamoto *et al*. 2011) and has also been identified in the O-chain of several *E. coli* strains. In addition to viosamine, the A-clade is the only clade to have rhamnose, a deoxy-sugar found as a component of the O-antigen of several bacteria, such as *S. enterica* (Samuel and Reeves 2003), in the N-glycans of archaea (Kaminski and Eichler 2014) and plant cell-wall polysaccharides. Interestingly, the A-clade follows the plant way instead of the microbial pathway to synthetize rhamnose (Parakkottil Chothi *et al*. 2010). The precursor for both these sugars is UDP-d-Glc, which is the only UDP-sugar for which the A-clade does not appear to have a dedicated biosynthetic machinery, thus relying on its host.

Except for *Moumouvirus australiensis*, B-clade members have two rare amino sugars, d-Qui2NAc and d-Fuc2NAc, whose biosynthetic pathways are closely interconnected. d-Fuc2NAc was found in the LPS of *Pseudomonas aeruginosa* O5 (Burrows *et al*. 1996) and in the O-Chain of the marine bacterium *Pseudoalteromonas agrivorans* (Perepelov *et al*. 2000). d-Qui2NAc was also found in the O-chain of Gram-negative bacteria, such as *Rizobium etli CE3* (D'Haeze *et al*. 2007) and *Pseudomonas aeruginosa* O10 (Knirel *et al*. 1986).

C-clade viruses, unlike B-clade, decorate their fibrils with L-RhaNAc and l-Qui2NAc that is also a component of *Moumouvirus australiensis* fibrils and is shared with *Cotonvirus japonicus*. l-Qui2NAc is a component of the O-antigen of several pathogenic bacteria, such as *Yersinia enterocolitica* serotype O11, 23 and O11, 24 (Marsden *et al*. 1994), *Vibrio cholerae* O37 (Kneidinger *et al*. 2003) and *Proteous penneri* 26 (Shashkov *et al*. 1998) and has also been found in the capsular polysaccharide (CPS) of *Bacteroides fragilis* NCT 9343 and ATCC 23 745 (Kasper *et al*. 1983). Rhamnosamine is a rare sugar found only in *Proteous vulgaris* TG 155 O-antigen (Kondakova *et al*. 2003), *E. coli* O3 LPS (Jaan and Jann 1968) and *Vibrio vulnificus* CPS (Reddy *et al*. 1993).

Finally, *Moumouvirus australiensis* (outsider of the B-clade) along with *Cotonvirus japonicus* and *Tupanviruses* are characterized by another rare amino sugar, d-diNacBac, which has also been found in pathogenic bacteria (Morrison and Imperiali 2014) as the reducing sugar of N-linked and O-linked glycoproteins of *C. jejuni* and *Neisseria gonorrhoeae*, respectively. It is also a component of the O-Chain of *Pseudomonas reactans* and *Vibrio cholerae*, and of the CPS of *Alteromonas*(Perepelov *et al*.^2000^, Aydanian *et al*.^2011^).

The precursor of the rare amino-sugars characteristic of B- and C-clades, *Cotonvirus japonicus* and *Tupanviruses* is UDP-d-GlcNAc, for which all clades possess the biosynthetic pathway. Consequently, in contrast to the A-clade, they could be completely independent from their host for synthesis of their glycans, which could possibly extend the range of hosts that they can replicate in.

Chemical analyses of the fibrils in *Tupanvirus* and *Cotonvirus japonicus* were not performed and gene expression data are also lacking. However, our results showing conservation of the biosynthetic pathways for Qui2N and diNAcBac in *Cotonvirus* suggest that its fibrils could be decorated with the corresponding glycans, while those in *Tupanviruses* could be composed of diNAcBac, Fuc2N/Qui2N and GlcA. The UDP-GlcNAc biosynthetic pathway identified in the A-, B- and C-clades is also conserved in *Cotonvirus japonicus* and in *Tupanviruses* (Table 1). Thus, further studies are needed to determine whether their fibrils could be glycosylated using virus-encoded enzymes.

Why are giant viruses covered by rare amino sugars and why are these different depending on the clade? The presence of a highly glycosylated fibril layer for all members of proposed *Megavirinae* isolated so far suggests an essential role played by these glycans in the environment. Regarding the first question, we think that the presence of bacterial-like sugars on the surface of giant viruses can be considered as a strategy to mimic the bacteria that the amoeba feeds on, thus facilitating competition with other parasites for the same host in the natural environment. We could also speculate that they play an important role in the physiology of these viruses. In fact, it has been established that glycans play a crucial role in the adhesion process to the host cell, while they do not affect dramatically the viral replication even if they appear to have a fitness cost (Boyer *et al*. 2011b). In this context, amino sugars are essential because their physiochemical properties enable them to create a highly viscous or sticky surface (Salton 1965), which is suitable for adhesion on cell surfaces. In addition, glycans constitute a protective barrier both against the natural environment in which they must propagate and the enzymes of the host cell. Finally, these viruses are targets of viral infection by virophages, which stick to the giant virus fibrils (Duponchel and Fischer 2019; La Scola *et al*. 2008), allowing them to be transported along into the host cell. Some virophages, like Sputnik (La Scola *et al*. 2008), are deleterious to the giant virus, while others, like Zamilon (Jeudy *et al*. 2020), appear to be commensal. Evolving different sugar compositions for fibril glycosylation could thus impair virophage interaction with the fibrils and be advantageous against pathogenic virophages.

The answer to the second question comes from the organization of the glycogenes in hot-spot mutation areas that could be essential to introduce variability in glycan composition. Because fibril glycans play a pivotal role in the interaction with the host cell, an arms race is taking place between the host and the giant viruses, but also among giant viruses that compete for the same host in the natural environment. Having a flexible toolbox for glycan synthesis could reflect the need to continuously adjust the set of glycans decorating the capsids to trigger increased phagocytosis and outcompete other parasites. It is tempting to compare giant virus glycosylation clusters with antibiotic resistance cassettes in bacteria. The fact that this complex glycosylation machinery is lost in laboratory conditions, as was shown for M4 (Boyer *et al*. 2011a), also suggests that there must be a fitness cost for such complex glycan synthesis, again echoing what is observed for antibiotic resistance genes in bacteria.

## Conclusions

This in-depth study of glycosylation in the proposed *Megavirinae* subfamily revealed that giant DNA viruses are different from other eukaryotic viruses in several aspects. First, they possess a complex glycosylation machinery consisting of clusters of six to 33 genes responsible for the formation of glycans constituting their fibrils. This results in glycosylated fibrils with sugars different from those found in the host and from those of other eukaryotic viruses. Giant viruses produce rare amino sugars, yet there is a clade-specific glycosylation trend, although some exceptions could occur, as is shown for *Moumouvirus australiensis* that could be in the process of evolving a new glycosylation cassette (losing and/or gaining new glycosylation genes).

This variegated glycosylation between the different clades could be the result of adaptation to the host they infect and/or competition with other viruses and bacteria and could also affect their ability to be infected by virophages.

Studying the glycobiology of these giant DNA viruses is important as it may reveal new enzymes encoded by genes of unknown function located within these clusters and could provide clues to the origin of the various elements of their glycosylation machinery, the glycosylation machinery of their ancestor that may predate the radiation of eukaryotes, and ultimately lead to advances for the glycobiology discipline in general. Finally, this work could be considered as a pilot study, which can be extended to other giant and large DNA viruses, such as the atypical *Pandoraviruses*, *Pithoviruses* and *Molliviruses*.

## Supplementary Material

uqac002_Supplemental_File

## References

[bib2] Abergel C , Joëlle R-T, Richard G et al. Virus-encoded aminoacyl-tRNA synthetases: structural and functional characterization of Mimivirus TyrRS and MetRS. J Virol. 2007;81:12406–17. 17855524 10.1128/JVI.01107-07 PMC2169003

[bib1] Abergel C , Legendre M, Claverie J-M. The rapidly expanding universe of giant viruses: Mimivirus, Pandoravirus, Pithovirus and Mollivirus. FEMS Microbiol Rev. 2015;39:779–96. 26391910 10.1093/femsre/fuv037

[bib3] Abrahão J , Lorena S, Ludmila SS et al. Tailed giant Tupanvirus possesses the most complete translational apparatus of the known virosphere. Nat Commun. 2018;9:749. 29487281 10.1038/s41467-018-03168-1 PMC5829246

[bib4] Akey DL , Li S, Konwerski JR et al. A new structural form in the SAM/Metal-Dependent O–Methyltransferase family: Myce from mycinamicin biosynthetic pathway. J Mol Biol. 2011;413:438–50. 21884704 10.1016/j.jmb.2011.08.040 PMC3193595

[bib5] Armougom F , Sébastien M, Olivier P et al. Expresso: automatic incorporation of structural information in multiple sequence alignments using 3D-Coffee. Nucleic Acids Res. 2006;34:W604–08. 16845081 10.1093/nar/gkl092 PMC1538866

[bib7] Aydanian A , Tang Li, Morris JG et al. Genetic diversity of O-antigen biosynthesis regions in Vibrio cholerae. Appl Environ Microbiol. 2011;77:2247–53. 21317260 10.1128/AEM.01663-10 PMC3067440

[bib8] Bagdonaite I , Wandall HH. Global aspects of viral glycosylation. Glycobiology. 2018;28:443–67. 29579213 10.1093/glycob/cwy021 PMC7108637

[bib9] Boyer M , Azza S, Barrassi L et al. Mimivirus shows dramatic genome reduction after intraamoebal culture. Proc Natl Acad Sci. 2011a;108:10296–301. 21646533 10.1073/pnas.1101118108 PMC3121840

[bib10] Boyer M , Saïd A, Lina B et al. Mimivirus shows dramatic genome reduction after intraamoebal culture. Proc Natl Acad Sci. 2011b;108:10296–301. 21646533 10.1073/pnas.1101118108 PMC3121840

[bib11] Brussaard CP , Steven WW, Frede T et al. Global-scale processes with a nanoscale drive: the role of marine viruses. ISME J. 2008;2:575–8. 18385772 10.1038/ismej.2008.31

[bib12] Burrows LL , Charter DF, Lam JS. Molecular characterization of the Pseudomonas aeruginosa serotype O5 (PAO1) B-band lipopolysaccharide gene cluster. 1996;22:481–95. 10.1046/j.1365-2958.1996.1351503.x 8939432

[bib13] Burrows LL , Urbanic RV, Lam JS. Functional conservation of the polysaccharide biosynthetic protein WbpM and its homologues in Pseudomonas aeruginosa and other medically significant bacteria. Infect Immun. 2000;68:931–36. 10639466 10.1128/iai.68.2.931-936.2000 PMC97225

[bib14] Byrne D , Grzela R, Lartigue A et al. The polyadenylation site of Mimivirus transcripts obeys a stringent ‘hairpin rule’. Genome Res. 2009;19:1233–42. 19403753 10.1101/gr.091561.109 PMC2704430

[bib15] D'Haeze W , Leoff C, Freshour G et al. Rhizobium etli CE3 bacteroid lipopolysaccharides are structurally similar but not identical to those produced by cultured CE3 bacteria. J Biol Chem. 2007;282:17101–13. 17420254 10.1074/jbc.M611669200

[bib17] De Castro C , Molinaro A, Piacente F et al. Structure of N-linked oligosaccharides attached to chlorovirus PBCV-1 major capsid protein reveals unusual class of complex N-glycans. Proc Natl Acad Sci. 2013;110:13956–60. 23918378 10.1073/pnas.1313005110 PMC3752267

[bib16] De Castro C , Parrilli M, Holst O et al. Microbe-associated molecular patterns in innate immunity. Methods Enzymol. 2010;480:89–115. 20816206 10.1016/S0076-6879(10)80005-9

[bib18] De Castro C , Speciale I, Duncan G et al. N-linked glycans of chloroviruses sharing a core architecture without precedent. Angew Chem Int Ed. 2016;55:654–8. 10.1002/anie.201509150 PMC4836869 26582281

[bib6] Defne A , Legendre M, Seltzer V et al. Distant Mimivirus relative with a larger genome highlights the fundamental features of Megaviridae. Proc Natl Acad Sci. 2011;108:17486–91. 21987820 10.1073/pnas.1110889108 PMC3198346

[bib19] Duponchel S , Fischer MG. Viva lavidaviruses! Five features of virophages that parasitize giant DNA viruses. PLoS Pathog. 2019;15:e1007592. 30897185 10.1371/journal.ppat.1007592 PMC6428243

[bib20] Fischer MG , Allen MJ, Wilson WH et al. Giant virus with a remarkable complement of genes infects marine zooplankton. Proc Natl Acad Sci. 2010;107:19508–13. 20974979 10.1073/pnas.1007615107 PMC2984142

[bib21] Gallot-Lavallée L , Blanc G, Claverie J-M. Comparative genomics of chrysochromulina ericina virus and other microalga-infecting large DNA viruses highlights their intricate evolutionary relationship with the established mimiviridae family. J Virol. 2017;91. 10.1128/JVI.00230-17 PMC5487555 28446675

[bib22] Gouet P , Robert X, Courcelle E. ESPript/ENDscript: extracting and rendering sequence and 3D information from atomic structures of proteins. Nucleic Acids Res. 2003;31:3320–3. 12824317 10.1093/nar/gkg556 PMC168963

[bib23] Gronow S , Brade H. Invited review: Lipopolysaccharide biosynthesis: which steps do bacteria need to survive?. J Endotoxin Res. 2001;7:3–23. 11521077

[bib24] Hildebrand A , Remmert M, Biegert A et al. Fast and accurate automatic structure prediction with HHpred. Proteins Struct Funct Bioinf. 2009;77:128–32. 10.1002/prot.22499 19626712

[bib25] Jaan B , Jann K. 2-Amino-2, 6-Dideoxy-L-Mannose (L-Rhamnosamine) isolated from the lipopolysaccharide of escherichia coli 03: k2ab(L):H2. 1968;5:173–7. 10.1111/j.1432-1033.1968.tb00354.x 4875435

[bib26] Jeudy S , Abergel C, Claverie J-M et al. Translation in giant viruses: a unique mixture of bacterial and eukaryotic termination schemes. PLos Genet. 2012;8:e1003122. 23271980 10.1371/journal.pgen.1003122 PMC3521657

[bib27] Jeudy S , Bertaux L, Alempic J-M et al. Exploration of the propagation of transpovirons within mimiviridae reveals a unique example of commensalism in the viral world. ISME J. 2020;14:727–39. 31822788 10.1038/s41396-019-0565-y PMC7031253

[bib28] Jumper J , Evans R, Pritzel A et al. Highly accurate protein structure prediction with alphafold. Nature. 2021;596:583–9. 34265844 10.1038/s41586-021-03819-2 PMC8371605

[bib29] Kaminski L , Eichler J. Haloferax volcanii N-glycosylation: delineating the pathway of dTDP-rhamnose biosynthesis. PLoS One. 2014;9:e97441. 24831810 10.1371/journal.pone.0097441 PMC4022621

[bib30] Kasper DL , Weintraub A, Lindberg AA et al. Capsular polysaccharides and lipopolysaccharides from two bacteroides fragilis reference strains: chemical and immunochemical characterization. J Bacteriol. 1983;153:991–7. 6185469 10.1128/jb.153.2.991-997.1983 PMC221723

[bib31] King JD , Poon KKH, Webb NA et al. The structural basis for catalytic function of GMD and RMD, two closely related enzymes from the GDP-d-rhamnose biosynthesis pathway. FEBS J. 2009;276:2686–700. 19459932 10.1111/j.1742-4658.2009.06993.x PMC4381037

[bib32] Klose T , Herbst DA, Zhu H et al. A Mimivirus enzyme that participates in viral entry. Structure. 2015;23:1058–65. 25982526 10.1016/j.str.2015.03.023 PMC4456301

[bib33] Kneidinger B , Larocque S, Brisson J-R et al. Biosynthesis of 2-acetamido-2,6-dideoxy-L-hexoses in bacteria follows a pattern distinct from those of the pathways of 6-deoxy-L-hexoses. Biochem J. 2003;371:989–95. 12575896 10.1042/BJ20030099 PMC1223350

[bib34] Knirel YA , Vinogradov EV, Shashkov AS et al. Somatic antigens of Pseudomonas aeruginosa. The structure of O-specific polysaccharide chains of P. aeruginosa O10 (Lányi) lipopolysaccharides. Eur J Biochem. 1986;157:129–38. 3086090 10.1111/j.1432-1033.1986.tb09648.x

[bib35] Kondakova AN , Kolodziejska K, Zych K et al. Structure of the N-acetyl-L-rhamnosamine-containing O-polysaccharide of Proteus vulgaris TG 155 from a new proteus serogroup, O55. Carbohydr Res. 2003;338:1999–2004. 14499576 10.1016/s0008-6215(03)00327-6

[bib36] La Scola B , Audic S, Robert C et al. A giant virus in amoebae. Science. 2003;299:2033. 12663918 10.1126/science.1081867

[bib37] La Scola B , Desnues C, Pagnier I et al. The virophage as a unique parasite of the giant Mimivirus. Nature. 2008;455:100–4. 18690211 10.1038/nature07218

[bib38] Legendre M , Audic S, Poirot O et al. mRNA deep sequencing reveals 75 new genes and a complex transcriptional landscape in Mimivirus. Genome Res. 2010;20:664–74. 20360389 10.1101/gr.102582.109 PMC2860168

[bib39] Li T et al. *In* *vitro* biosynthesis and chemical identification of UDP- *N* -acetyl-d-quinovosamine (UDP-d-QuiNAc). J Biol Chem. 2014;289:18110–20. 24817117 10.1074/jbc.M114.555862 PMC4140256

[bib40] Lönngren J , Svensson S. Mass spectrometry in structural analysis of natural carbohydrates. Adv Carbohydr Chem Biochem. 1974;29:41–106.

[bib41] Lu R , Zhao X, Li J et al. Genomic characterisation and epidemiology of 2019 novel coronavirus: implications for virus origins and receptor binding. Lancet North Am Ed. 2020;395:565–74. 10.1016/S0140-6736(20)30251-8 PMC7159086 32007145

[bib42] Marsden BJ , Bundle DR, Perry MB. Serological and structural relationships between Escherichia coli O:98 and Yersinia enterocolitica O:11,23 and O:11,24 lipopolysaccharide O-antigens. Biochem Cell Biol. 1994;72:163–8. 7530967 10.1139/o94-024

[bib43] Morrison MJ , Imperiali B. The renaissance of bacillosamine and its derivatives: pathway characterization and implications in pathogenicity. Biochemistry. 2014;53:624–38. 24383882 10.1021/bi401546r PMC3951908

[bib44] Noel E , Notaro A, Speciale I et al. Chlorovirus PBCV-1 multidomain protein A111/114R has three glycosyltransferase functions involved in the synthesis of atypical N-Glycans. Viruses. 2021;13:87. 33435207 10.3390/v13010087 PMC7826918

[bib45] Notaro A , Couté Y, Belmudes L et al. Expanding the occurrence of polysaccharides to the viral world: the case of Mimivirus. Angew Chem Int Ed Engl. 2021;60:19897–904. 34241943 10.1002/anie.202106671 PMC8456856

[bib46] Olivier NB , Imperiali B. Crystal structure and catalytic mechanism of PglD from *Campylobacter**jejuni*. J Biol Chem. 2008;283:27937–46. 18667421 10.1074/jbc.M801207200 PMC2562079

[bib47] Parakkottil Chothi M , Duncan GA, Armirotti A et al. Identification of an L-rhamnose synthetic pathway in two nucleocytoplasmic large DNA viruses. J Virol. 2010;84:8829–38. 20538863 10.1128/JVI.00770-10 PMC2918987

[bib48] Perepelov AV , Senchenkova S'N, Shashkov AS et al. First application of triflic acid for selective cleavage of glycosidic linkages in structural studies of a bacterial polysaccharide from Pseudoalteromonas sp. KMM 634 †. J Chem Soc, Perkin Trans 1. 2000;1:363–6.

[bib49] Philippe N , Legendre M, Doutre G et al. Pandoraviruses: Amoeba viruses with genomes up to 2.5 MB reaching that of Parasitic Eukaryotes. Science. 2013;341:281–6. 23869018 10.1126/science.1239181

[bib50] Piacente F , Bernardi C, Marin M et al. Characterization of a UDP-N-acetylglucosamine biosynthetic pathway encoded by the giant DNA virus Mimivirus. Glycobiology. 2014a;24:51–61. 24107487 10.1093/glycob/cwt089

[bib51] Piacente F , De Castro C, Jeudy S et al. Giant virus Megavirus chilensis encodes the biosynthetic pathway for uncommon acetamido sugars. J Biol Chem. 2014b;289:24428–39. 25035429 10.1074/jbc.M114.588947 PMC4148869

[bib52] Piacente F , de Castro C, Jeudy S et al. The rare sugar N-acetylated viosamine is a major component of Mimivirus fibers. J Biol Chem. 2017a;292:7385–94. 28314774 10.1074/jbc.M117.783217 PMC5418040

[bib54] Piacente F , Gaglianone M, Elena Laugieri M et al. The autonomous glycosylation of large DNA viruses. Int J Mol Sci. 2015;16:29315–28. 26690138 10.3390/ijms161226169 PMC4691112

[bib53] Piacente F , Marin M, Molinaro A et al. Giant DNA virus Mimivirus encodes pathway for biosynthesis of unusual sugar 4-amino-4,6-dideoxy-D-glucose (Viosamine). J Biol Chem. 2012;287:3009–18. 22157758 10.1074/jbc.M111.314559 PMC3270958

[bib55] Raoult D . The 1.2-Megabase genome sequence of Mimivirus. Science. 2004;306:1344–50. 15486256 10.1126/science.1101485

[bib56] Reddy GP , Hayat U, Bush CA et al. Capsular polysaccharide structure of a clinical isolate of Vibrio vulnificus strain BO62316 determined by heteronuclear NMR spectroscopy and high-performance anion-exchange chromatography. Anal Biochem. 1993;214:106–15. 8250211 10.1006/abio.1993.1463

[bib57] Renesto P , Abergel C, Decloquement P et al. Mimivirus giant particles incorporate a large fraction of anonymous and unique gene products. J Virol. 2006;80:11678–85. 16971431 10.1128/JVI.00940-06 PMC1642625

[bib59] Riegert AS , Thoden JB, Schoenhofen IC et al. Structural and biochemical investigation of PglF from *Campylobacter**jejuni* reveals a new mechanism for a member of the short chain dehydrogenase/reductase superfamily. Biochemistry. 2017;56:6030–40. 29053280 10.1021/acs.biochem.7b00910 PMC6211297

[bib58] Riegert AS , Young NM, Watson DC et al. Structure of the external aldimine form of PglE, an aminotransferase required for *n*, *n* ’-diacetylbacillosamine biosynthesis: structure of the Aminotransferase PglE'. Protein Sci. 2015;24:1609–16. 26178292 10.1002/pro.2745 PMC4594660

[bib60] Salton MRJ . Chemistry and function of amino sugars and derivatives. Annu Rev Biochem. 1965;34:143–74. 14321166 10.1146/annurev.bi.34.070165.001043

[bib61] Samuel G , Reeves P. Biosynthesis of O-antigens: genes and pathways involved in nucleotide sugar precursor synthesis and O-antigen assembly. Carbohydr Res. 2003;338:2503–19. 14670712 10.1016/j.carres.2003.07.009

[bib62] Schulz F , Yutin N, Ivanova NN et al. Giant viruses with an expanded complement of translation system components. Science. 2017;356:82–5. 28386012 10.1126/science.aal4657

[bib63] Shashkov AS , Paramonov NA, Veremeychenko SP et al. Somatic antigens of Pseudomonads: structure of the O-specific polysaccharide of Pseudomonas fluorescens biovar B, strain IMV 247. Carbohydr Res. 1998;306:297–303. 9691454 10.1016/s0008-6215(97)10048-9

[bib64] Speciale I , Laugieri ME, Noel E et al. Chlorovirus PBCV-1 protein A064R has three of the transferase activities necessary to synthesize its capsid protein N-linked glycans. Proc Natl Acad Sci. 2020;117:28735. 33139538 10.1073/pnas.2016626117 PMC7682578

[bib65] Suhre K , Audic S, Claverie JM. Mimivirus gene promoters exhibit an unprecedented conservation among all eukaryotes. Proc Natl Acad Sci. 2005;102:14689–93. 16203998 10.1073/pnas.0506465102 PMC1239944

[bib66] Suttle CA . Viruses in the sea. Nature. 2005;437:356–61. 16163346 10.1038/nature04160

[bib67] Szymanski CM , Yao R, Ewing CP et al. Evidence for a system of general protein glycosylation in Campylobacter jejuni. Mol Microbiol. 1999;32:1022–30. 10361304 10.1046/j.1365-2958.1999.01415.x

[bib68] Takahashi H , Fukaya S, Song C et al. Morphological and taxonomic properties of the newly isolated Cotonvirus japonicus, a new lineage of the subfamily Megavirinae. J Virol. 2021;95:Jvi0091921. 10.1128/JVI.00919-21 PMC8387033 34191583

[bib69] Yamamoto M , Ohnishi-Kameyama M, Nguyen CL et al. Identification of genes involved in the glycosylation of modified viosamine of flagellins in Pseudomonas syringae by mass spectrometry. Genes. 2011;2:788–803. 24710292 10.3390/genes2040788 PMC3927599

[bib70] Zhao X , Chen H, Wang H. Glycans of SARS-CoV-2 spike protein in virus infection and antibody production. 2021;8:629873. 10.3389/fmolb.2021.629873 PMC8076860 33928117

