# Supplementary material for: Giant viruses of the Megavirinae subfamily possess biosynthetic pathways to produce rare bacterial-like sugars in a clade-specific manner
Source: Microlife. 2022 Apr 6;3:uqac002. doi: 10.1093/femsml/uqac002 (PMC10117803; doi:10.1093/femsml/uqac002)
Supplement: uqac002_Supplemental_File [file uqac002_supplemental_file.docx]

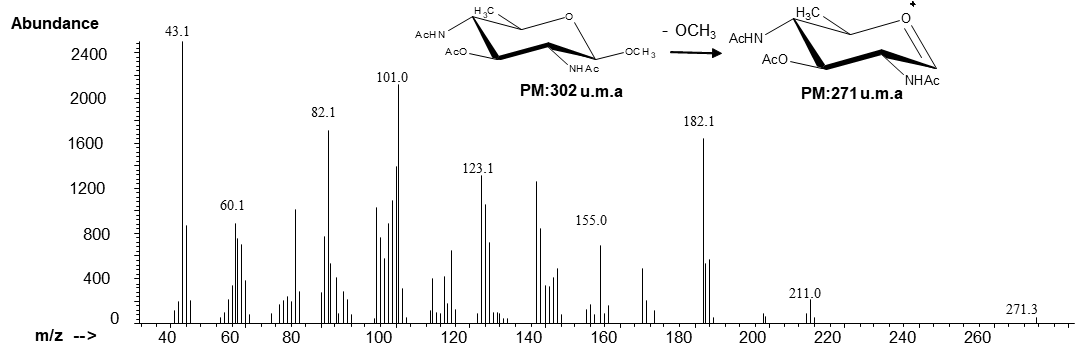
**Fig. S1. EI-MS spectrum of the diNAcBac (RT 19 min), a sugar component of *Moumouvirus australiensis* fibrils.** The fragment at m/z 271 is consistent with the oxonium ion of a six-deoxy-sugar with two amino functions.


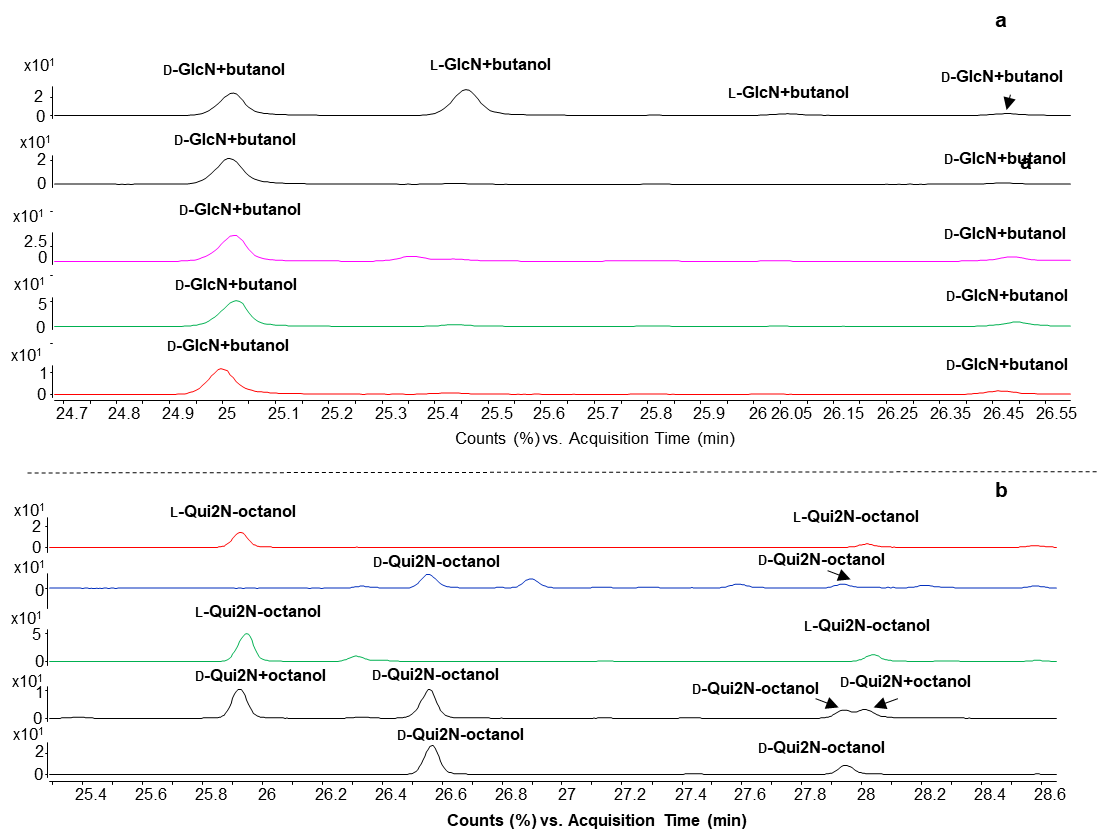
**Fig. S2. Determination of the absolute configuration of a) GlcN in *Mimivirus* (violet), *Megavirus chilensis* (green) and *Moumouvirus australiensis* (red); b) Qui2N in *Megavirus chilensis* (green), *Moumouvirus australiensis* (red) and *Moumouvirus maliensis* (blue) via GC-MS**. The absolute configuration of GlcN and Qui2N was established by the comparison with opportune standards (reported in black). In detail, it was always used a racemic mixture of D-sugar +- octanol/butanol standard (black) and a standard of D-sugar-+/- 2-octanol/butanol (black).


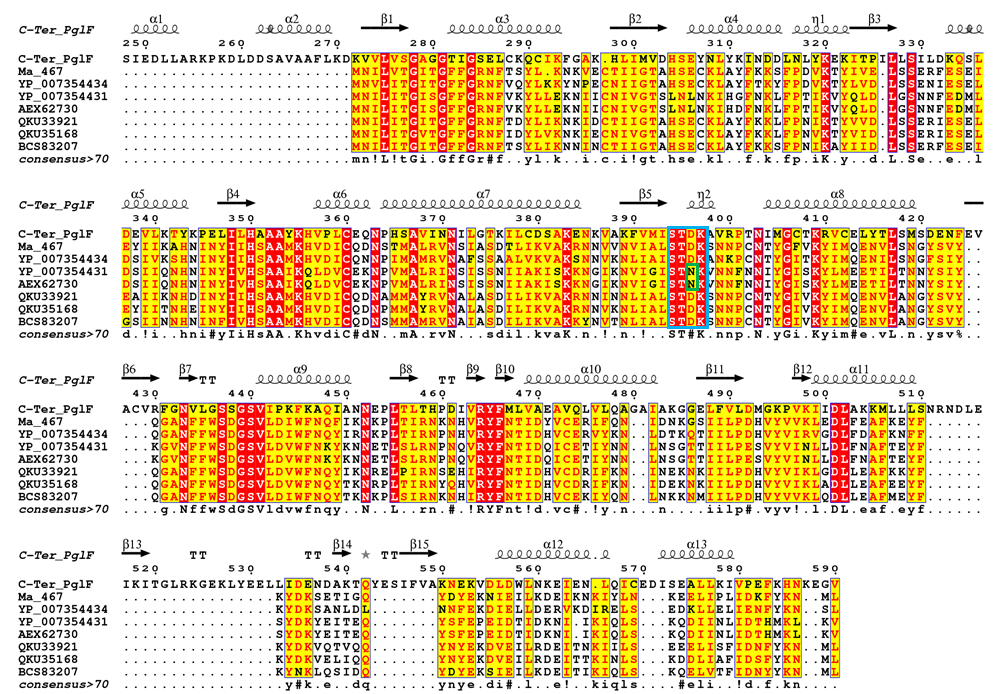


**Fig. S3.** Multiple alignment of the C-Terminal catalytic domain (248-590) of PglF (PDB 5BJU), *Moumouvirus australiensis* Ma467 (AVL94853), *Moumouvirus* gp 461 (YP_007354434) and gp464 (YP_007354431), *Moumouvirus monve*, mvR525 (AEX62730), *Tupanvirus soda lake* (QKU35168) and *Tupanvirus deep ocean* (QKU33921), and Cotonvirus japonicus (BCS83207). The blue rectangle indicates the residues of the catalytic site. The green rectangle indicates the mutation D396N in gp464 and mvR525. This multiple alignment confirms that all catalytic residues are conserved in *Moumouvirus australiensis,* *Tupanviruses* and C*otonvirus japonicus*, suggesting that these proteins could catalyse the dehydration of UDP-D-N-Acetyl-GlcN into UDP-4-keto-2,4,6-trideoxy-D-glucose (Fig. 4). The multiple alignment was performed with Expresso Server (Armougom et al., 2006) and the picture was done with ESPript (Gouet et al., 2003).


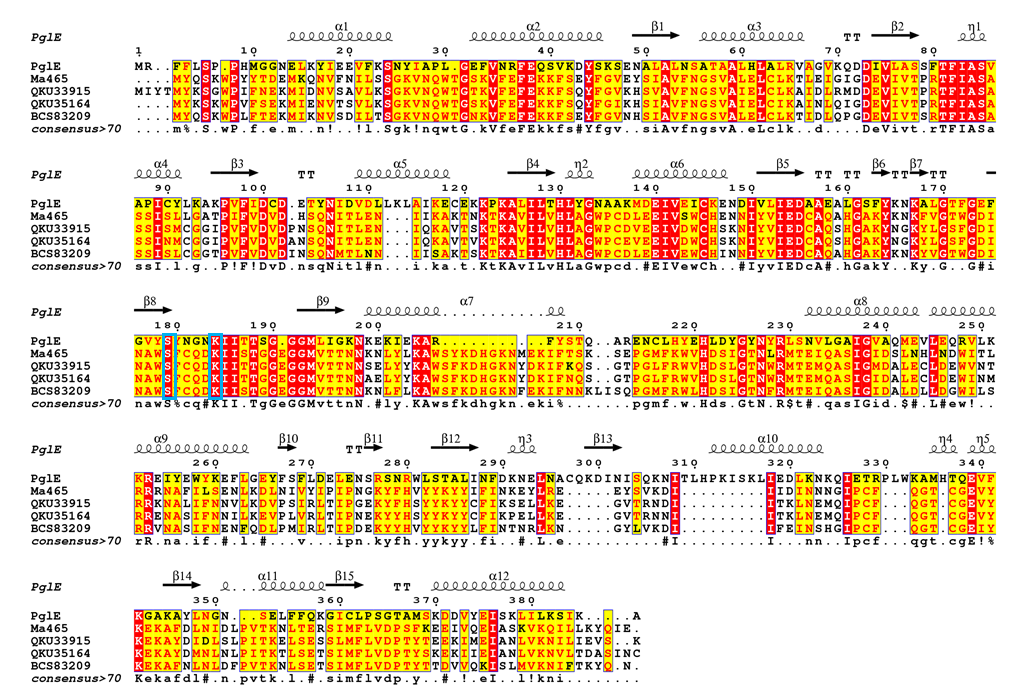


**Fig. S4.**  Multiple alignment of the PglE (PDB 1O61), Ma465 (AVL94851) of *Moumouvirus australiensis*, its homolog in *Tupanvirus deep ocean* (QKU33915), *Tupanvirus soda lake* (QKU35164*)* and *Cotonvirus japonicus* (BCS83209). The blue rectangles mark the residues involved in the catalytic activity. Based on its homology with PglE (PDB#1O61), we propose Ma465 could be a pyridoxal phosphate (PLP) aminotransferase catalysing the second reaction of the diNAcBac pathway, namely the transfer of an amino group from L-glutamate to the C-4 of the UDP-2-acetamido-4-keto-2,4,6-trideoxy-D-glucose, leading to the formation of UDP-2-acetamido-4-amino-2,4,6-trideoxy-D-glucose (Fig. 4). This multiple alignment of Ma465 and its orthologs with PglE as reference structure, confirms that all residues involved in PLP binding are conserved, suggesting it could play the same role in the viral diNAcBac pathway. The multiple alignment was performed with Expresso Server (Armougom et al., 2006) and the picture was done with ESPript (Gouet et al., 2003).


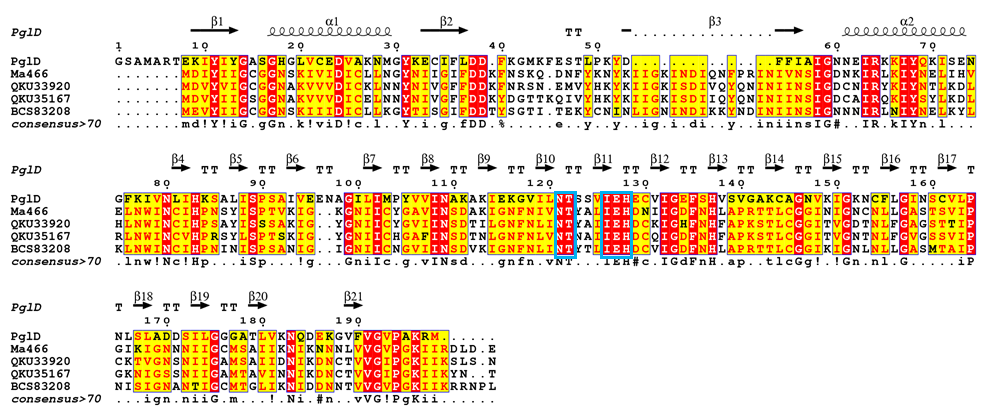
**Fig. S5.** Multiple alignment of PglD (PDB 3BSS), Ma466 (AVL94852) of *Moumouvirus australiensis*, its homolog in *Tupanvirus deep ocean* (QKU33920), *Tupanvirus soda lake* (QKU35167) and *Cotonvirus japonicus* (BCS83208). The blue rectangles indicate the residues involved in the catalytic activity (Olivier and Imperiali, 2008). This analysis confirms the conservation of all residues involved in the catalysis (Olivier and Imperiali 2008), thus proposing Ma466 is involved in the last reaction step of the diNAcBac production, namely the acetylation of the amino function at position four. The multiple alignment was produced using Expresso Server (Armougom et al., 2006) and the picture was done with ESPript (Gouet et al., 2003).


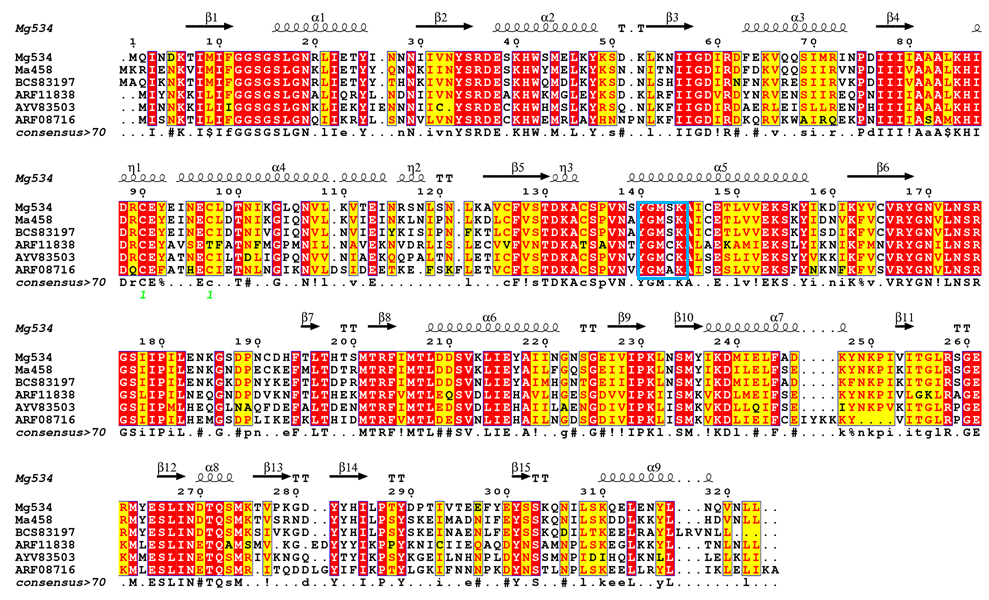
**Fig. S6.** Multiple alignment of Mg534 (PDB 4TQG_A) with its orthologs in *Moumouvirus australiensis* Ma458 (AVL94844), *Cotonvirus japonicus* (BCS83197), *Klosneuvirus* KNV1 (ARF11838), *Hyperinovirus* (AYV83503), and *Catovirus* CTV1 (ARF08716). The blue rectangle marks the catalytic triad containing the Y140XXXK144 motif, conserved in other short-chain dehydrogenases/oxidoreductase (SDR) enzymes. The multiple alignment was performed with Expresso Server (Armougom et al., 2006) and the picture was done with ESPript (Gouet et al., 2003).


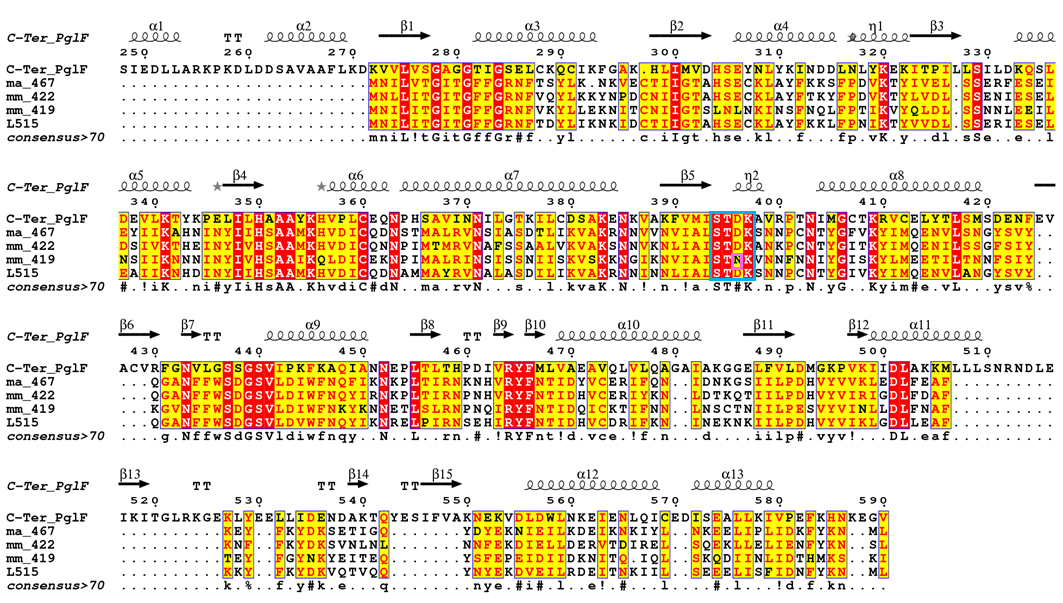
**Fig. S7.** Multiple alignment of the C-Terminal catalytic domain (248-590) of PglF (PDB 5BJU), *Moumouvirus australiensis* Ma467 (AVL94853), *Moumouvirus maliensis* Mm422 (QGR53991) and Mm419 (QGR53988) and *Tupanvirus soda lake* (QKU35168). The blue rectangle indicates the residues of the catalytic site. The pink rectangle indicates the mutation D150N in Mm419 that suppresses the catalytic activity. The multiple alignment was performed with Expresso Server (Armougom et al., 2006) and the picture was done with ESPript (Gouet et al., 2003).


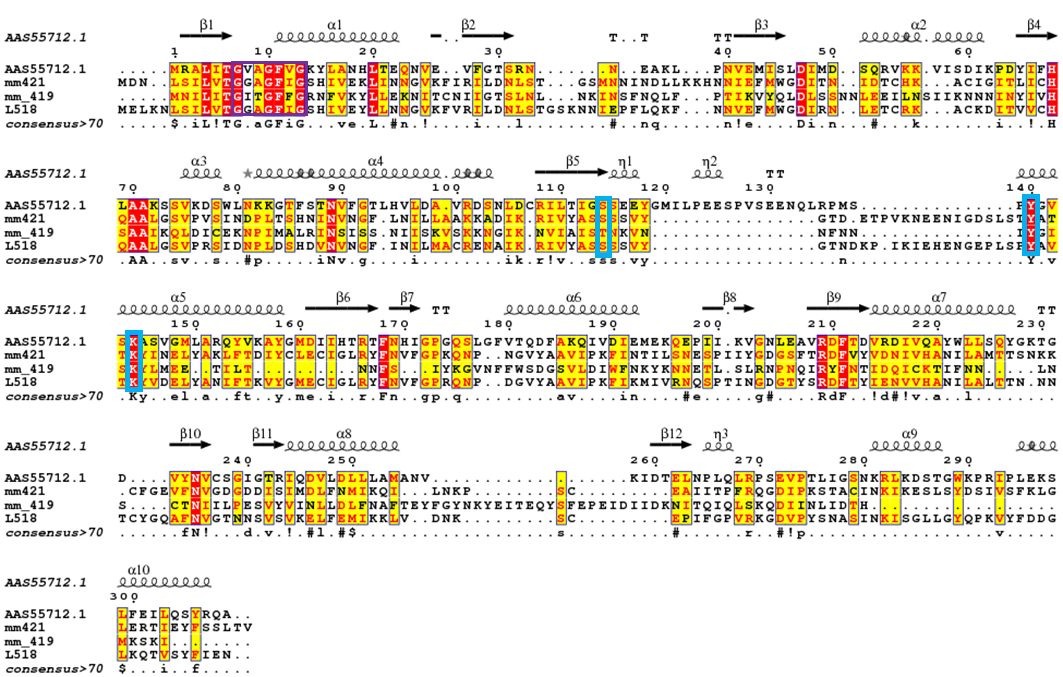
**Fig.S8.** Multiple alignment of 4-reductase of *A. thermoaerophilus* (AAS55712.1), *Moumouvirus maliensis* Mm421 (QGR53990) and Mm419 (QGR53988) and *Tupanvirus soda lake* L518 (QKU3517). The blue rectangles indicate the catalytic triad. The violet rectangle indicates the NADP binding site. The multiple alignment was performed with Expresso Server (Armougom et al., 2006) and the picture was done with ESPript (Gouet et al., 2003).

**Fig.S9. a) Proposed biosynthetic pathways for UDP-D-glucuronic acid in Tupanvirus deep ocean.** The *Burkholderia cepacia* reference protein (2Y0C) is in red and the corresponding *Tupanvirus deep ocean protein* (QKU33925) is in light grey and underlined (64% coverage and 30% identity). **b)** Multiple alignment of UDP-6-glucose dehydrogenase of *Burkholderia cepacia* (2Y0C), *Tupanvirus deep ocean* R520 (QKU33925), *Tupanvirus soda lake* (QKU35172), *Catovirus* (ARF08707), *Klosneuvirus* (ARF11434) and *Cafeteria roenbergensis* virus (YP_003969874). The R520 enzyme exhibits up to 50% identity on the entire protein sequence with its orthologs. The pink rectangle shows the glycine-rich consensus sequence, typical of the dehydrogenase enzymes. The blue rectangles indicate the residues involved in the catalysis. The multiple alignment was performed with Expresso Server (Armougom et al., 2006) and the picture was done with ESPript (Gouet et al., 2003).
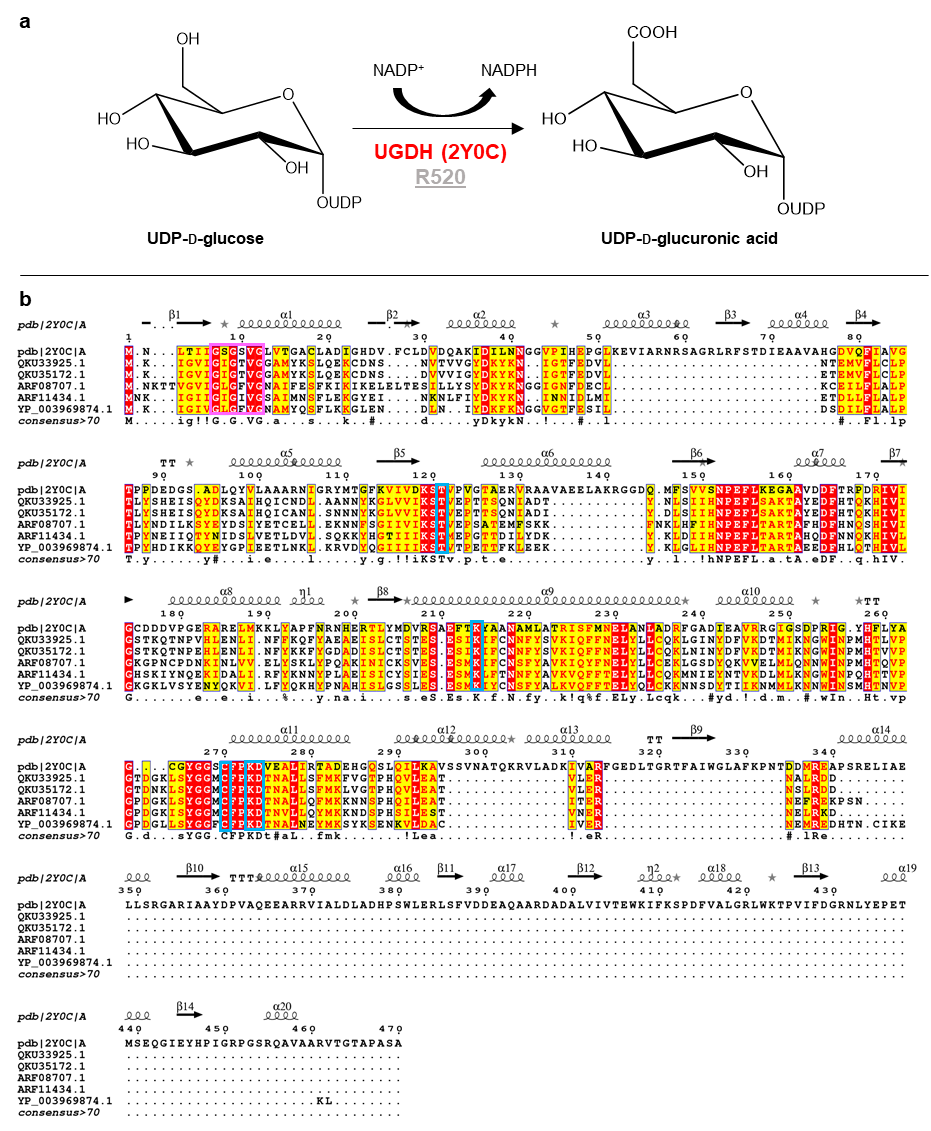


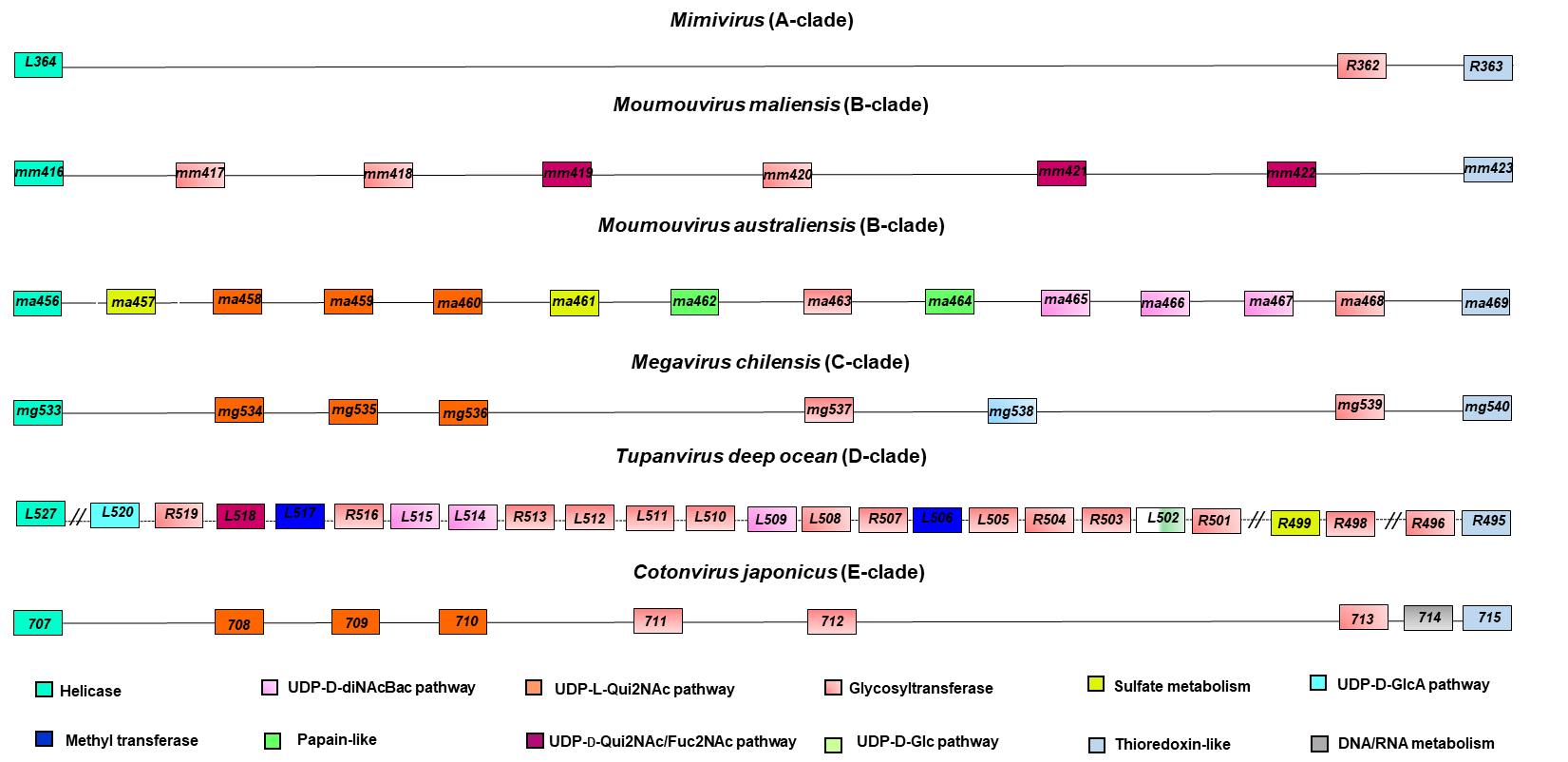
**Fig. S10. Schematic representation of the genomic region between the conserved helicase (light grey) and thioredoxin-like (light yellow) in:** Mimivirus (A-clade), Moumouvirus australiensis (B-clade), Moumouvirus maliensis (B-clade), Megavirus chilensis (C-clade), Cotonvirus japonicus and Tupanvirus deep ocean. The function of each gene is color coded (explained in the legend) and explained in detail in the text. The // indicates that one gene is missing in the representation. ORFs are not drawn to scale.


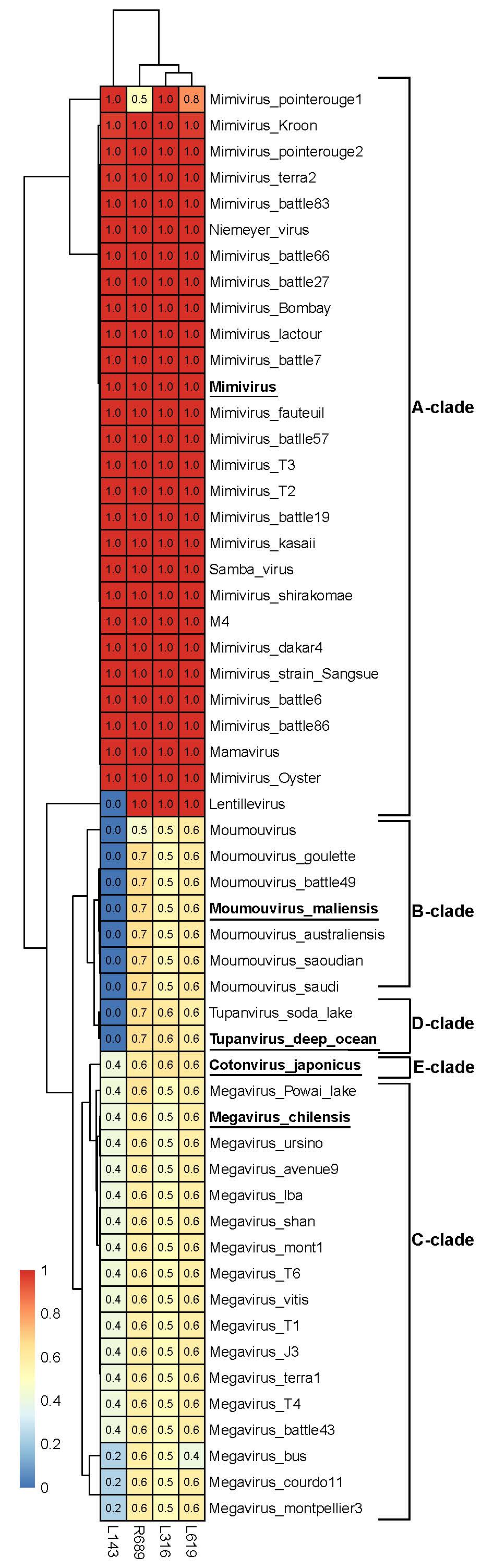


**Figure S11. Heatmap of the conservation level of all the enzymes involved in the UDP-D-GlcNAc production in the *Megavirinae* family.** We have included L143 enzyme that could be responsible of the glucosamine pyruvylation at the level of the UDP-sugar or after the transfer to its acceptor. The score of each protein in each genome is shown with values ranging from 1 to 0 (red to blue). The prototype of each clade is in bold and underlined.

**
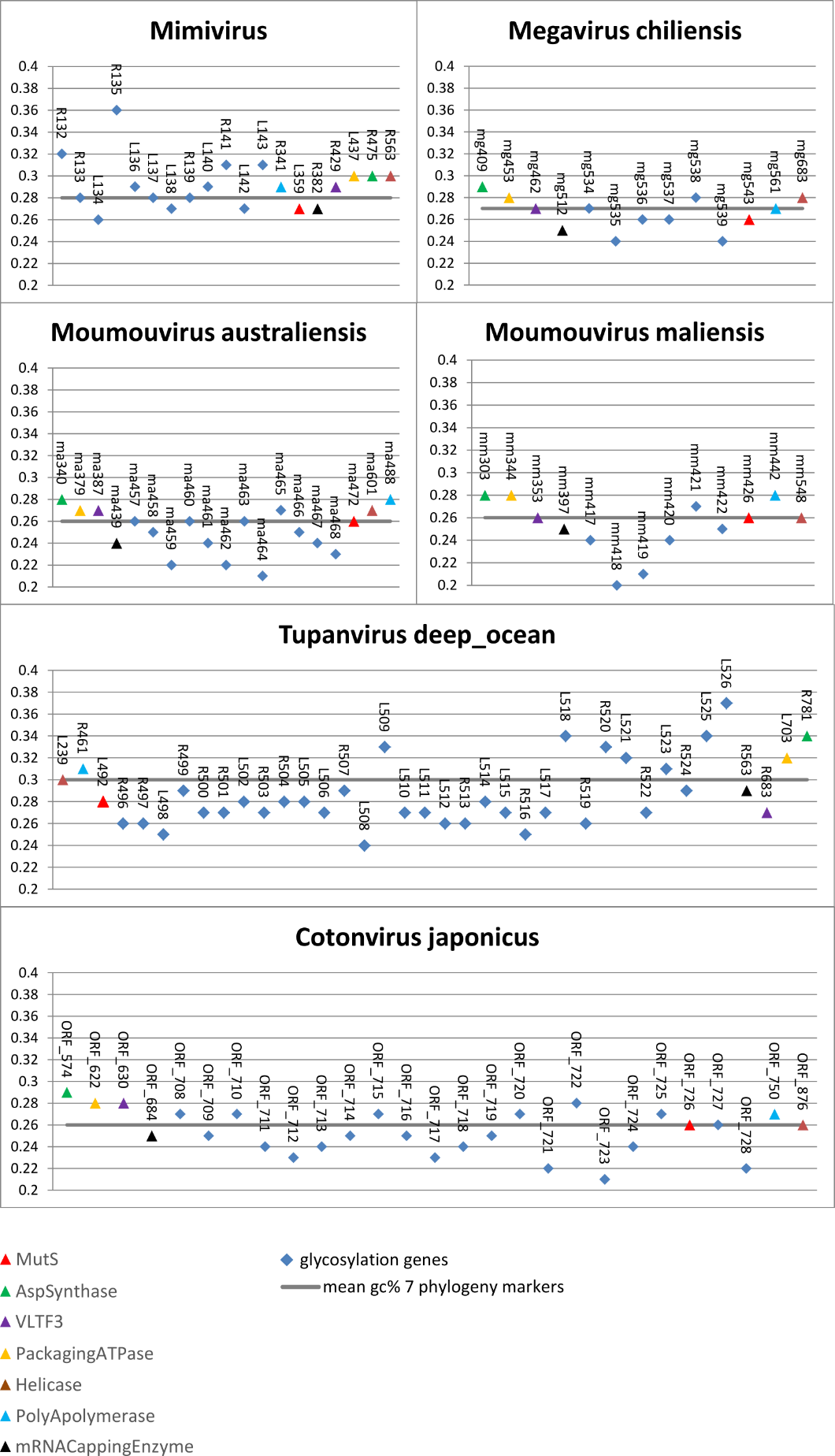
**

**Fig. S12. GCcontent of the glycosylatioon genes clusters of *Mimivrus*, *Megavirus chilensis*, *Moumouvirus autraliensis*, *Moumouvirus maliensis*, *Tupanvirus deep ocean* and *Cotonvirus japonicus*.** The glyco-genes (in light blue) seem to have the same GC content of the rest of the genomes. 7 genes markers (Asp Synthase, Helicase, Mrna Capping Enzyme, MutS, Packaging ATPase, PolyA polymerase and VLTF3) as representative of the GC% content of the whole genome (colore code according to the figure legend). The GCcontent of these genes was calculated using geecee (Emboss package v6.6.0, https://www.bioinformatics.nl/cgi-bin/emboss/geecee).

**Table S1: Genomes accession numbers used to build the heatmaps.** NCBI accession number of the complete genome sequences used in the tBlastn search to build the heatmaps of the level of conservation of the proteins involved in the glycans production of *Megavirinae* fibrils.

| Source | Accession | Clade |
| --- | --- | --- |
| Mimivirus | NC_014649 [NC_014649](https://www.ncbi.nlm.nih.gov/nuccore/NC_014649.1) | A |
| Mimivirus_batlle57 | CWJZ00000000 | A |
| Mimivirus_battle19 | CWJX00000000 | A |
| Mimivirus_battle27 | CWJS00000000 | A |
| Mimivirus_battle66 | CWKA00000000 | A |
| Mimivirus_battle6 | CWJU00000000 | A |
| Mimivirus_battle7 | CWJW00000000 | A |
| Mimivirus_battle83 | CWKG00000000 | A |
| Mimivirus_battle86.fa | CWKD00000000 | A |
| Mimivirus_Bombay.fa | KU761889 | A |
| Mimivirus_dakar4 | CWKF00000000 | A |
| Mimivirus_fauteuil | LN871163.1 | A |
| Mimivirus_kasaii | AP017644.1 | A |
| Mimivirus_Kroon | KM982402 | A |
| Mimivirus_lactour | CXOL00000000 | A |
| Mimivirus_m4 | JN036606 | A |
| Mimivirus_Oyster | KM982401 | A |
| Mimivirus_pointerouge1 | LN871174 | A |
| Mimivirus_pointerouge2 | LN871172.1 | A |
| Mimivirus_shirakomae | AP017645 | A |
| Mimivirus_T2 | CXOT00000000 | A |
| Mimivirus_T3 | CXOR00000000 | A |
| Mimivirus_terra2 | NC_023639. | A |
| Niemeyer_virus | KT599914.1 | A |
| Samba_virus | KF959826.2 | A |
| Lentillevirus | AFYC01000005 | A |
| Hirudovirus_strain_Sangsue | KF493731.1 | A |
| Mamavirus | JF801956 | A |
| Moumouvirus_battle49 | LN871171. | B |
| Moumouvirus australensis | MG807320 | B |
| Moumouvirus | MG807320 | B |
| Moumouvirus_goulette | KC008572. | B |
| Moumouvirus_saoudian | CXOQ00000000 | B |
| Moumouvirus_maliensis | MK978772 | B |
| Moumouvirus_saudi | KY110734 | B |
| Megavirus_battle43 | LN868256. | C |
| Megavirus_avenue9 | LN867403.1 | C |
| Megavirus_bus | LN868539 | C |
| Megavirus_curdo7 | ASM296631v1 | C |
| Megavirus_chilensis | NC_016072. | C |
| Megavirus_courdo11 | JX975216 | C |
| Megavirus_J3 | CWIN00000000 | C |
| Megavirus_lba | JX885207 | C |
| Megavirus_mont1 | CWIO00000000 | C |
| Megavirus_montpellier3 | LN868518.1 | C |
| Megavirus_Powai_lake | KU877344. | C |
| Megavirus_shan | LN868520 | C |
| Megavirus_T1 | LN868526 | C |
| Megavirus_T4 | LN869537 | C |
| Megavirus_T6 | CWJQ00000000 | C |
| Megavirus_vitis | MG807319 | C |
| Megavirus_terra | KF527229 | C |
| Megavirus_ursino.fa | CWJY00000000 | C |
| Cotonvirus japonicus | AP024483 | Cotonvirus |
| Tupanvirus soda lake | KY523104 | Tupanvirus |
| Tupanvirus deep ocean | MF405918 | Tupanvirus |

| Source | Protein | Accession number |
| --- | --- | --- |
| Mimivirus | L619 | YP_003987136 |
| Mimivirus | L316 | YP_00398619 |
| Mimivirus | R689 | YP_03987216 |
| Mimivirus | R132 | YP_003986624 |
| Mimivirus | R133 | YP_003986625 |
| Mimivirus | L134 | YP_003986626 |
| Mimivirus | R135 | YP_003986627 |
| Mimivirus | L136 | YP_003986628 |
| Mimivirus | L137 | YP_003986629 |
| Mimivirus | L138 | YP_003986630 |
| Mimivirus | R139 | YP_003986631 |
| Mimivirus | L140 | YP_003986632 |
| Mimivirus | R141 | YP_003986633 |
| Mimivirus | L142 | YP_003986634 |
| Mimivirus | L143 | YP_003986635 |
| Moumouvirus australensis | Ma456 | AVL94842 |
| Moumouvirus australensis | Ma457 | AVL94843 |
| Moumouvirus australensis | Ma458 | AVL94844 |
| Moumouvirus australensis | Ma459 | AVL94845 |
| Moumouvirus australensis | Ma460 | AVL94846 |
| Moumouvirus australensis | Ma461 | AVL94847 |
| Moumouvirus australensis | Ma463 | AVL94849 |
| Moumouvirus australensis | Ma465 | AVL94851 |
| Moumouvirus australensis | Ma466 | AVL94852 |
| Moumouvirus australensis | Ma467 | AVL94853 |
| Moumouvirus australensis | Ma468 | AVL94854 |
| Moumouvirus maliensis | Mm416 | QGR53985 |
| Moumouvirus maliensis | Mm417 | QGR53986 |
| Moumouvirus maliensis | Mm418 | QGR53987 |
| Moumouvirus maliensis | Mm419 | QGR53988 |
| Moumouvirus maliensis | Mm420 | QGR53989 |
| Moumouvirus maliensis | Mm421 | QGR53990 |
| Moumouvirus maliensis | Mm422 | QGR53991 |

**Table S2. Protein accession number of the protein sequence used to build the heatmaps**. NCBI accession number of the protein used in the tblastn search to build the heatmaps.

**Table S3. *Mimivirus* (A-clade) glycosyltransferases share strong identity.** For each GT protein is reported the NCBI Accession number, the protein length and the % of identity with other GT localized inside the 12-gene cluster. R139 is the only GT that doesn’t have similarity with the other GT of the cluster.

| **GT-Protein** | **Accession** | **Length (aa)** | **Similarity between *Mimivirus* GTs** | | |
| --- | --- | --- | --- | --- | --- |
|  |  |  | **% ID** | **% Cover** | **Protein** |
| L137 | YP_003986629 | 732 | 56.57 | 34 | C-Ter L142 |
|  |  |  | 49.64 | 37 | L140 |
| L138 | YP_003986630 | 883 | 52.78 | 28 | C-Ter L142 |
|  |  |  | 45.36 | 32 | L140 |
| R139 | YP_003986631 | 248 | - | - | - |
| L140 | YP_003986632 | 304 | 49.64 | 90 | L137 |
|  |  |  | 45.07 | 89 | L138 |
| L142 | YP_003986634 | 490 | 56.57 | 51 | N-Ter L137 |
|  |  |  | 52.57 | 51 | N-Ter-L138 |
